# Applications of synthetic biology in medical and pharmaceutical fields

**DOI:** 10.1038/s41392-023-01440-5

**Published:** 2023-05-11

**Authors:** Xu Yan, Xu Liu, Cuihuan Zhao, Guo-Qiang Chen

**Affiliations:** 1 School of Life Sciences, Tsinghua University, 100084 Beijing, China; 2 PhaBuilder Biotech Co. Ltd., Shunyi District, Zhaoquan Ying, 101309 Beijing, China; 3 Center for Synthetic and Systems Biology, Tsinghua University, 100084 Beijing, China; 4 MOE Key Lab for Industrial Biocatalysis, Dept Chemical Engineering, Tsinghua University, 100084 Beijing, China

**Keywords:** Biotechnology, Nanobiotechnology

## Abstract

Synthetic biology aims to design or assemble existing bioparts or bio-components for useful bioproperties. During the past decades, progresses have been made to build delicate biocircuits, standardized biological building blocks and to develop various genomic/metabolic engineering tools and approaches. Medical and pharmaceutical demands have also pushed the development of synthetic biology, including integration of heterologous pathways into designer cells to efficiently produce medical agents, enhanced yields of natural products in cell growth media to equal or higher than that of the extracts from plants or fungi, constructions of novel genetic circuits for tumor targeting, controllable releases of therapeutic agents in response to specific biomarkers to fight diseases such as diabetes and cancers. Besides, new strategies are developed to treat complex immune diseases, infectious diseases and metabolic disorders that are hard to cure via traditional approaches. In general, synthetic biology brings new capabilities to medical and pharmaceutical researches. This review summarizes the timeline of synthetic biology developments, the past and present of synthetic biology for microbial productions of pharmaceutics, engineered cells equipped with synthetic DNA circuits for diagnosis and therapies, live and auto-assemblied biomaterials for medical treatments, cell-free synthetic biology in medical and pharmaceutical fields, and DNA engineering approaches with potentials for biomedical applications.

## Introduction

The concept of synthetic biology was proposed in 1910s by Stephane Le Duc.^1^ In this field, research strategies have been changed from the description and analysis of biological events to design and manipulate desired signal/metabolic routes, similar to the already defined organic synthesis. Unlike organic synthesis successfully developed in the early 19^th^ century,^2^ synthetic biology is restricted by DNA, RNA and protein technology within the complexity of biological systems. Today, synthetic biology has been developed extensively. It becomes a multidisciplinary field aims to develop new biological parts, systems, or even individuals based on existing knowledge. Researchers can apply the engineering paradigm to produce predictable and robust systems with novel functionalities that do not exist in nature. Synthetic biology is tightly connected with many subjects including biotechnology, biomaterials and molecular biology, providing methodology and disciplines to these fields.

**Fig. 1:**
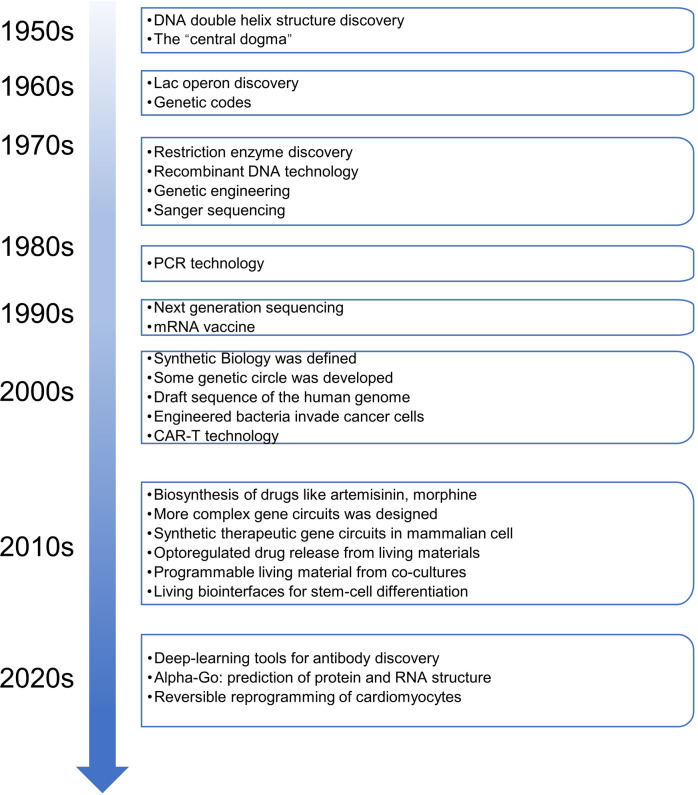
Timeline of major milestones in synthetic biology. The timeline begins at 1950s and expands to 2020s. Important events are listed in the right panels

The timeline of synthetic biology developments is summarized here (Fig. 1). In general, the history of synthetic biology can be divided into three stages. The initial stage was found across the 20th century. Although the simplest organisms such as virus particles, bacteria, archaea and fungi were hard to engineer in the 20th century, some achievements were still acquired in the early explorations including the synthesis of crystalline bovine insulin,^3^ chemical synthesis of DNA and RNA,^4^ decoding of genetic codes^5^ and the establishment of central dogma of molecular biology.^6^ Synthetic biology has been accumulating its strengths in this period, as knowledge of genome biology and molecular biology are developed rapidly at the end of the 20th century (Fig. 1).

The development stage begins in the 21^st^ century. In the first decade of the new millennium, synthetic biology is known to every biological researcher to include inventions of bioswitches,^7^ gene circuits based on quorum sensing signals,^8^ yeast cell-factory for amorphadiene synthesis^9^ (Table 1), BioBrick standardized assembly^10^ and the iGEM conferences^11^ (Fig. 1). Two principles in synthetic biology designs have been considered in this stage including bottle-up^12^ and top-down^13^ ones, referring to the de novo creations of artificial lives by assembling basic biological molecules and engineering natural-existed cells to meet actual demands, respectively. However, most circuits are well-designed but still not enough for producing complex metabolites or sensing multiple signals, especially the applications are not well prepared for medical and pharmaceutical usages. Anyhow, synthetic biology is gradually becoming a most topical area, on the eve of rapid developments.

**Table 1:** Applications and yields of biosynthesized pharmaceuticals

	Application	Classification	Production host	Titer (g/L)	Year	Ref.
Artemisic acid	A precursor of anti-malarial drug artemisinin	Terpenoids	*Saccharomyces cerevisiae*	25	2013	^23^
Thebaine	Pain management and palliative care	Alkaloids	*E. coli*/*Saccharomyces cerevisiae*	2.1 × 10^−3^/6.6 × 10^−5^	2016/2015	^323,324^
Hydrocodone	Pain management and palliative care	Alkaloids	*E. coli/Saccharomyces cerevisiae*	4 × 10^−5^/3 × 10^−7^	2016/2015	^323,324^
Codeine	Treat severe pain	Alkaloids	*E. coli*	304 µg L^−1^ OD^−1^	2019	^396^
Sitagliptin	Increased insulin secretion	Amines	In vitro	N.A.^a^	2010	^337^
Ginsenoside Rh2	Cancer prevention and therapy	Terpenoids	*Saccharomyces cerevisiae*	2.2	2019	^317^
Ginsenoside compound K	Increased resistance to stress and aging	Terpenoids	*Saccharomyces cerevisiae*	1.4 × 10^−3^ / 1.17/5.0	2014/2020/2021	^318,397,398^
Guaia-6,10(14)-diene	A precursor of kidney cancer drug Englerin A	Terpenoids	*Saccharomyces cerevisiae*	0.8	2020	^399^
Taxadiene	A precursor of cancer drug Taxol	Terpenoids	*E. coli / Saccharomyces cerevisiae*	1.0 / 8.7 × 10^−3^	2010/2008	^315,400^
Adenosylcobalamin (vitamin B12)	Vital cofactor for human	Corrinoids	*E. coli*	307 µg g^−1^ DCW^b^	2018	^401^
Baicalein	Neuroprotective agent	Flavonoids	*E. coli*	0.02	2019	^402^
Miltiradiene	A precursor of cardiovascular diseases drug tanshinone	Terpenoids	*Saccharomyces cerevisiae*	0.3 / 3.5	2012/2020	^403,404^
Catharanthine	A precursor of anti-cancer drug vinblastine and vincristine	Alkaloids	*Saccharomyces cerevisiae*	2.7 × 10^−5^	2022	^405^
Breviscapine	Treat cardio- and cerebrovascular diseases	Flavonoids	*Saccharomyces cerevisiae*	0.2	2018	^406^
Scopolamine	Treat neuromuscular disorders	Alkaloids	*Saccharomyces cerevisiae*	6 × 10^−2^	2020	^407^
(S)-tetrahydropalmatine	Use as an analgesic and anxiolytic drug	Alkaloids	*Saccharomyces cerevisiae*	3.6 × 10^−6^	2021	^328^
Cannabigerolic acid	A precursor of various cannabinoids; reduce pain without hallucination	Alkaloids	*Saccharomyces cerevisiae*	0.1	2019	^327^
Triptolide	Treatment of rheumatoid arthritis	Terpenoids	*Saccharomyces cerevisiae*	30.5 μg g^−1^	2020	^408^
Psilocybin	Treatment of addiction, depression and post-traumatic stress disorder.	Amino acid derivatives	*E. coli / Saccharomyces cerevisiae*	1.2 / 0.6	2019 / 2020	^330,331^
Monacolin J	A precursor for simvastatin (Zocor), an important drug for treating hypercholesterolemia.	Polyketides	*Aspergillus terreus*	4.7	2017	^409^
Acarbose	Clinically used to treat patients with type 2 diabetes	oligosaccharide	*Actinoplanes sp*.	7.4	2020	^410^
α-Tocoterienol	Natural vitamin E, used as a valuable supplementation	Terpenoids	*Saccharomyces cerevisiae*	0.3	2020	^411^
Avermectin B1a	Widely used in the field of animal health, agriculture and human health	Polyketides	*Streptomyces avermitilis*	6.4	2010	^412^
Carnosic acid	Potent antioxidant and anticancer agents	Terpenoids	*Saccharomyces cerevisiae*	1 × 10^−3^	2016	^413^
Noscapine	Anticancer drug	Alkaloids	*Saccharomyces cerevisiae*	2.2 × 10^−3^	2018	^414^
Farnesene	Widely used in industry, a precursor of vitamin E	Terpenoids	*Saccharomyces cerevisiae*	55.4	2022	^415^
(−)-Deoxypodophyllotoxin	A precursor to anti-cancer drug etoposide	Alkaloids	*Nicotiana benthamiana*	4.3 mg/g dry plant weight	2019	^416^
Dencichine	Promote aggregation of platelets	Amino acid derivatives	*E. coli*	1.29	2022	^334^

^a^*N.A.* not applicable

^b^*DCW* the abbreviation of dry cell mass

The fast-growing stage begins from the 2010s, the emergences of genome editing technologies especially CRISPR/Cas9,^14^ low-cost DNA synthesis,^15^ next-generation DNA sequencing^16^ and high-throughput screening methods,^17^ workflows of design-build-test-learn (DBTL)^18^ and progresses in engineering biology^19^ (Fig. 1), have allowed synthetic biology to enter a fast-growing period,^20^ both in the lab-scale discoveries and industry-scale productions. Typically, Venter et al. assembled an artificial chromosome of *Mycoplasma mycoides* and transplanted it to *M. capricolum* to create new living cells.^21^ Besides, new methods have accelerated the discovery and engineering of metabolite biosynthesis pathways, microbial artemisinic acid synthesis has been made possible,^22,23^ which is the first industrialized plant metabolite produced by microbial cells. To realize the ultimate goal of design bio-systems similar to design electronic or mechanical systems, this is just the beginning. More efforts are needed to generate complex and stable biocircuits for various applications in the present of synthetic biology.

Besides scientists, investors also have realized the potentials in this field. Financial investments help establish synthetic-biology-related companies encouraged by the prediction that the global market of synthetic biology valued 9.5 billion dollars by 2021, including synthetic biology products (e.g., BioBrick parts, synthetic cells, biosynthesized chemicals) and enabling technologies (e.g., DNA synthesis, gene editing),^24^ they are expected to reach 37 billion dollars by 2026. Most investments focus on medical applications.^25^ Scientists and capital market are all optimistic about the future.

Started from chemical biosynthesis, synthetic biology has been expanded to cover areas in medical treatments, pharmaceutical developments, chemical engineering, food and agriculture, and environmental preservations. This paper focuses on the advances of synthetic biology in medical and pharmaceutical fields, including cell therapies, bacterial live diagnosis and therapeutics, production of therapeutic chemicals, nanotechnology and nanomaterial applications and targeted gene engineering.

## Genetic engineering of therapeutic chassis

### Engineered mammalian cells for medical applications

**Fig. 2:**
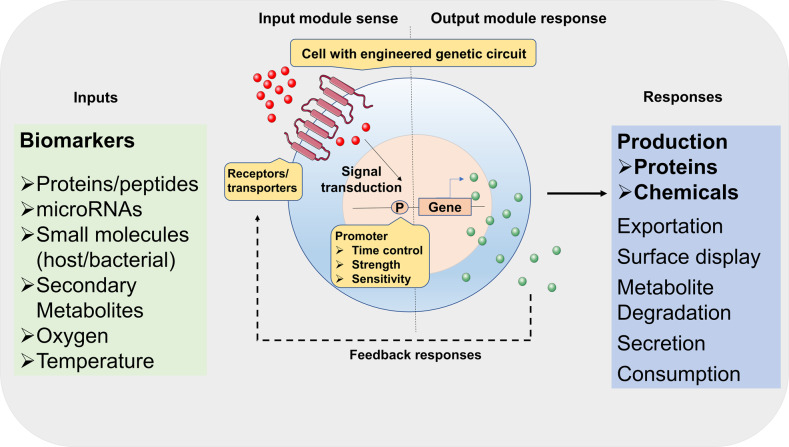
Development of smart living cells based on synthetic biology strategies. Smart cells can sense various environmental biomarkers, from chemicals to proteins. External signals are transducted into cells to trigger downstream responses. The products are also in the form of chemicals to proteins for customized demands. The sensing-reponsing system is endowing cells with new or enhanced abilities. P represents promoters

With the advances in synthetic biology, researchers created various novel therapies using living cell chassis rationally designed from existing signaling networks with new constructs for their purposes, including e.g., production of medical biomolecules, synthetic gene networks for sensing or diagnostics, and programmable organisms, to handle mechanisms underlying disease and related organism/individual events (Fig. 2). We highlighted here synthetic biology strategies in mammalian cell engineering for metabolic disorders, tissue engineering and cancer treatments, as well as approaches in cell therapy and the design of gene circuits.

#### Therapies based on chimeric antigen receptor (CAR)-T cells

**Fig. 3:**
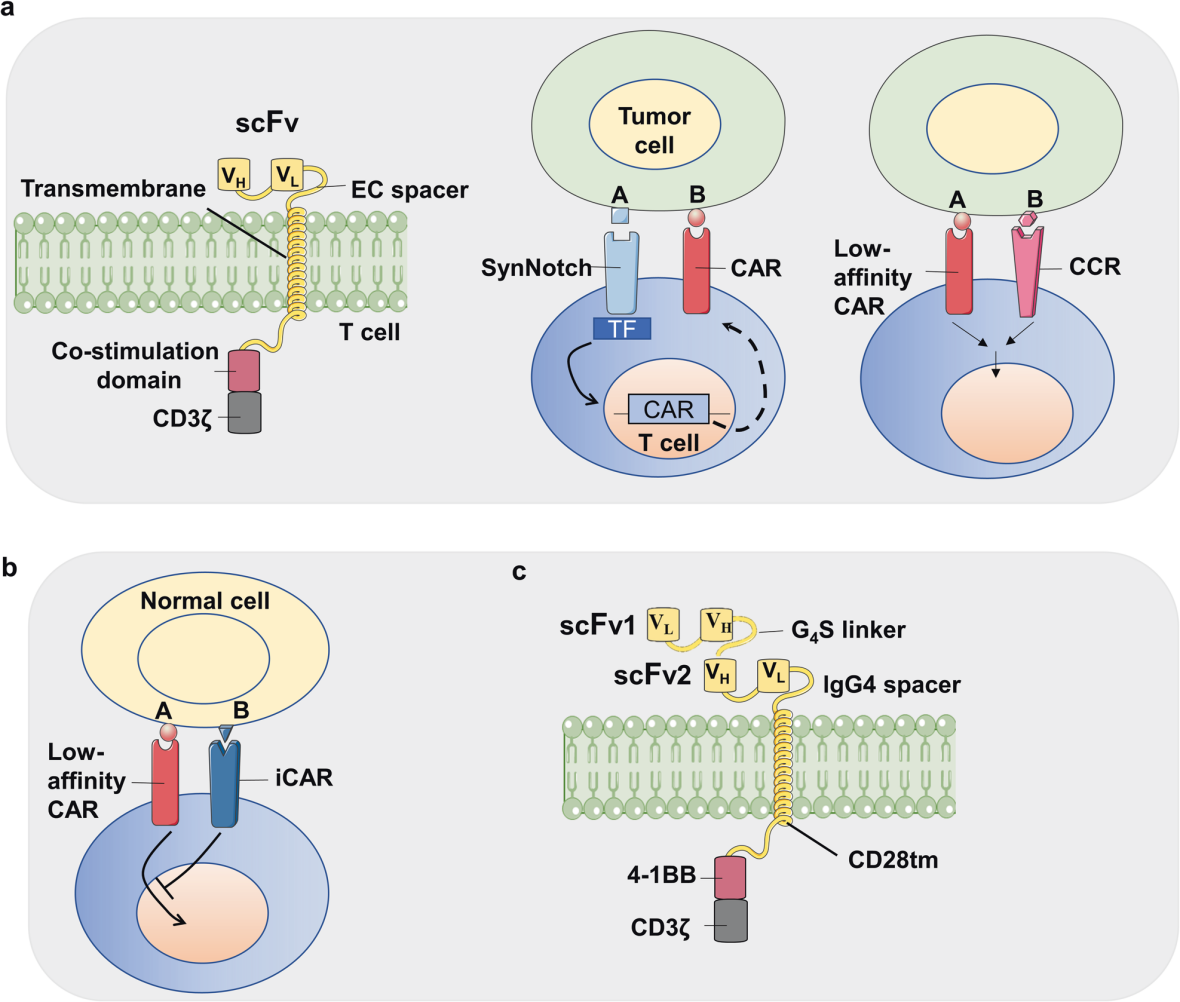
Synthetic biology in the designs of chimeric antigen receptors (CAR). **a** The AND gate used in artificial CARs. Three typical CARs i.e. Costimulation domain-based second-generation CAR, synNotch receptor-assisted CAR with multiple recognization mechanisms and chimeric costimulation receptor (CCR)-based CAR are exhibited from left to right. **b** The artificial CARs with inhibitory CAR (iCAR) system. The system can prevent recognizing self-antigens on somatic cells. **c** The artificial CARs sensing different tumor antigens. Two ScFvs recognizing different targets are tandemly fused, the engineered CAR can be triggered by multiple antigens. The figure is inspired by the paper^468^

CARs are engineered receptors containing both antigen-binding and T cell-activating domains. T cells are acquired from patients and engineered ex vivo to express a specific CAR, and followed by transferring into the original donor patient, where they eliminate cancer cells that surface-displayed the target antigen.^26^ CAR-T is a novel cell therapy began from 2000s.^27^ The first generation of CARs are single-chain variable fragments (scFv) targeting CD19.^28^ The development of artificial CARs comprises three generations. The first-generation CARs only contain a CD3ζ intracellular domain, while the second-generation CARs also possess a co-stimulatory domain, *e.g*., 4-1BB or CD3ζ (Fig. 3). Studies with the third-generation chimeric antigen receptors with multiple co-stimulatory signaling domains are also under investigation (Fig. 3).^29^ Because scFvs have the ability to recognize cell surface proteins, the targeting of tumors mediated via CAR-T cell is neither restricted nor dependent on antigen processing and presentation. CAR-T cells are therefore not limited to tumor escaping from MHC loss. For cancer immunotherapy, the main advantage of employing CAR-based methods is attributed to that the scFv derived from antibodies with affinities several orders of magnitude higher than conventional TCRs.^30^ In addition, CARs can target glycolipids, abnormal glycosylated proteins and conformational variants that cannot be easily recognized by TCRs. Based on clinical trial results, there is an increasing evidence that CAR-T cells have the ability to deliver powerful anti-tumor therapeutic effects, leading to the recent FDA approval of CAR-T therapies directed against the CD19 protein for the treatment of acute lymphoblastic leukemia (ALL) and large B-cell lymphoma (DLBCL).

In addition, CAR-T applications are stepping into commercialization. The first approved CAR T-cell therapy was Kymriah which is CD19-targeted for treating DLBCL developed by Novartis and University of Pennsylvania.^31^ DLBCL is a typical form of non-hodgkin lymphomas (NHL) that consist of 40% of total lymphomas.^32^ The FDA also approved Yescarta (axicabtagene ciloleucel) in 2017 for DLBCL treatments.^33^ In the clinical studies, patients with DLBCL were treated with the CD19-targeted CAR T-cells, with 25% partial responders and more than 50% complete responders.^34,35^ Durable responses of over two years were observed, indicating the therapeutic effects of the CAR-T cells. However, cytokine storm, an excessive release of pro-inflammatory cytokines, was observed in Yescarta treated patients (13%),^36^ indicating the safety needs to be improved.

The selection of target antigen is the determinant in CAR-T cell therapies.^37–39^ If CAR-T cells can recognize protein expressed on non-malignant cells, severe cell toxicities could occur with the off-target activities.^40^ The optimal target antigen is the one that is consistently expressed on the surface of cancer cells but not on the surface of normal cells.^37,41,42^ Multiple myeloma is hard to treat via chemicals or stem cell transplantation.^43,44^ CAR-T cell therapies are effective for multiple myeloma in preclinical studies.^45^ However, to date, no antigen has been characterized that is strongly and constantly expressed on multiple myeloma cells but not on somatic cells. Among the antigens used so far, a member of the TNF superfamily proteins, B cell maturation antigen (BCMA), is the most favorable candidate for a multiple myeloma cell-directed CAR-T therapy target.^42,46,47^ BCMA is expressed in cancer cells in almost all multiple myeloma patients, the expression of this antigen on somatic cells is limited to plasma cells and some kinds of B cells.^42,48^ BCMA was the first antigen for multiple myeloma to be used in a clinical trial via a CAR-T cell approach leading to systematic responses in patients with this cancer.^40,42,49^ Twelve patients received BCMA CAR-T cells in the dose-gradient clinical trial. Two patients treated with 9 × 10^6^ CAR-T cells/kg body weight were obtained with good remissions, though the treatment had toxicity related to cytokine storms.^49^ Many clinical trials investigating the safety and/or efficacy of anti-BCMA CAR-T cells are currently ongoing or finished.

Idecabtagene vicleucel (Abecma, also abbreviated as Ide-cel) is developed by Bristol-Myers Squibb, uses the anti-BCMA 11D5-3 scFv, the same as the 11D5-3-CD828Z CAR tested at the NCI.^49^ However, the co-stimulatory domain is different, the CAR used in idecabtagene vicleucel is delivered using a lentivirus vector and has a 4-1BB co-stimulatory domain instead of a CD28 one.^50^ In a multicenter phase I trial for idecabtagene vicleucel,^50,51^ the therapy is highly effective for treating multiple myeloma patients. A phase II trial named KarMMa, designed to further evaluate the safety and ability of idecabtagene vicleucel, is undergoing.^52^ The initial results of KarMMa demonstrates its deep, durable responses in heavily pretreated multiple myeloma patients.^52^ Efficacy and safety were reflected in early reports, supporting a favorable idecabtagene vicleucel clinical benefit-risk profile across the target dose range in primary clinical results.

#### Receptor engineering in medical therapies

SynNotch receptors are a class of artificially engineered receptors that are used in medical applications (Fig. 3).^53^ Notch receptors are transmembrane receptors participating in signal transductions,^54^ comprising an extracellular domain, a transmembrane and an intracellular domain.^55^ The transmembrane and intracellular domains are usually retained in synNotch architects,^56^ whereas the signal-input extracellular domain is engineered to sense scFvs and nanobodies,^57^ providing possibilities of recognizing agents to initiate signaling in living cells.

Also, the modular extracellular sensor architecture (MESA) was developed intending to detect extracellular free ligands^54,58^ based on the synNotch idea. MESA designs have two membrane proteins each containing an extracellular ligand-binding domain which senses the chemicals or proteins and can be a small molecule-binding domain or antibody based sensing module, a transmembrane domain and either an intracellular transcriptional factor with relasing ability from the complex, protease recognition sequence or a protease. After ligand binding to the extracellular domain, MESA receptors dimerize and induce an intracellular proteolytic cleavage that allows the transcriptional factor dissociate for downstream regulations. The method allows more flexible sensor designs without limiting to Notch receptors. This system has also been remade recently to signal transduction via a split protease^59^ or split transcriptional factor patterns.^60^ The synNotch design has been constructed with a series of receptors called synthetic intramembrane proteolysis receptors (SNIPRs) containing domains from other natural receptors other than mouse Notch protein that are also cleavable by endogenous proteases.^61^ Similiar to synNotch, SNIPRs bind to their antigens and function via dissociating a transcriptional factor to sense cell and immune factors.^62^ For synNotch, SNIPR and MESA, the choice of ligand-binding domains and transcriptional factor domains enables customization of both sensing (signal input) and function (signal output) steps when using the systems. SNIPR and MESA also enrich the available engineering tools for the artificial receptor-effectors. However, some limitations still remain such as high background signals, off-target effects, the immunogenicity from the murine Notch protein, the large size of artificial receptors and transcriptional regulators.^56,61,63^ Many efforts are needed to improve the system.

Receptor engineering applications are commonly related to CAR-T therapies. The receptors can be designed to target two specific antigens, one using the synNotch and the other via a traditional CAR. In preclinical models, T-cells engineered for targeting dual-antigen expressing cells are established.^64^ TEV protease can be fused to MESA receptors, cleaving the transcriptional factor off for functionalization.^58^ A humanized synthetic construct can reduce immunogenicity and minimize off-target effects. Zhu et al. constructed a framework for human SNIPRs with future applications in CAR-T therapies, preventing CAR-T cells from being activated via non-tumor signals.^61^ Besides the above synthetic receptors, based on the same idea, Engelowski et al. designed a synthetic cytokine receptor sensing nanobodies by the fusion of GFP/mCherry nanobodies to native IL-23 intracellular domains.^65^ Another receptor engineering strategy is to rewire receptor-transduced signals to novel effector genes. Using a scFv complementary to VEGF, the engineered receptor senses VEGF and released dCas9 protein, then the IL-2 expression are up-regulated. The system is successfully explored in Jurkat T cells.^58^

#### The HEK-β cells used for diabetes treatments

β-cells are existing in pancreatic islets that synthesize and secrete insulin.^66^ As the only site of insulin synthesis in mammals, β-cells sense blood glucose using a signal transduction pathway that comprises glycolysis and the stimulus-sensing-secretion coupling process.^67,68^ The secretion of insulin is consisted with the following steps. Blood glucose is transported into β-cells and metabolized via glycolysis inside the cell, resulting in cell membrane depolarization, energy generation and closing of K^+^ATP channels, which activates the calcium channel Cav1.3 to induce calcium influx with the secretion of insulin granules. The excessive blood-glucose concentration in diabetes patients is from the deficiency of insulin-producing β cells for type 1 diabetes, or from low insulin sensitivity of body cells for type 2 diabetes.^69^ Using a synthetic biology-based multiple screening approach, Xie et al. engineered human kidney cells HEK-293 to sense blood glucose levels for insulin secretion.^70^ The design combines automatic diagnosis and treatment in diabetes therapy. The researchers demonstrated that overexpression of Cav1.3 provided the pathway for constructing a β-cell-like glucose-sensing module in somatic cells.^70^ The combination of Cav1.3-controlled calcium and a synthetic Ca^2^^+^-inducible promoter allowed the monitoring of glucose levels using a tuned in vivo transcriptional response. After the construction of artificial HEK-293-β cells, the cell line HEK-293-β for glucose-response insulin production which maintained glucose homeostasis for over 3 weeks, via implanting the cells intraperitoneally to mice, also auto-corrected diabetic hyperglycemia within 3 days in T1D mice in this study.

The advantages of HEK-293-β cells are clear. Compared to primate pancreatic islets, HEK-293-β cells were adequately efficient in stabilizing postprandial glucose metabolism in T1D mice. Moreover, HEK-β cells are more easily for cultivation in vitro. It is expected that the engineered human cells have the prospect to be produced easily, cost-effectively and robustly, following current rules and regulations for pharmaceutical manufacturing, allowing the production of ready-to-use commercials with good properties for product purity, stability and quality. This highly innovative engineered cell raises the possibility that any cell type could be rationally reprogramming to achieve customized abilities such as blood glucose control.

#### The induced pluripotent stem cells (iPSCs) for medical applications

Synthetic biology also helps in generating human stem cells via overexpressing certain de-differentiation-related genes. One of the applications is the induced pluripotent stem cells. iPSCs are pluripotent stem cells generated from somatic cells.^71^ Pioneered by Yamanaka’s lab, the introduction of four transcriptional factors including Oct3/4, Sox2, c-Myc, and Klf4, resulted in changing fibroblasts to embryonic stem (ES)-like cells,^72^ which can re-differentiate into blood cells, bone cells or neurons for possible treatment of damages to various tissues and organs.^73^ iPSCs are not created using human embryos, circumvented ethical concerns in contrast with ES cells.^74^ Additionally, autologous somatic cell-derived iPSCs avoid immunological rejections.^75^

iPSCs are self-renewable with continuous subculture properties.^76^ The somatic cell samples from patients are induced into iPSCs able to serve as an unlimited repository for medical researches. The iPSC cell lines for Down’s syndrome and polycystic kidney disease are established.^77,78^ An project termed StemBANCC calls for collections of iPSC cell lines for drug screening.^79^ Various applications combined with therapeutic chemicals and iPSC cell lines are undergoing high-throughput drug screening and analysis.^80,81^

iPSCs are aimed to be used for tissue regeneration and therapy developments. Type O red blood cells can be derived from iPSCs to meet demands for blood transfusion.^82^ When cancer patients require large quantities of NK cells in immunotherapies, the cells can be manufactured using iPSCs to circumvent their low availabilities.^83^ The anti-aging effects of iPSCs are observed during mouse studies.^84^ The chemical-induced differentiation of iPSCs to cardiomyocytes has been commonly used.^85^ These iPSC-cardiomyocytes are recapitulated with genetic codes in patients whom they derived, allowing the establishment of models of long QT syndrome and ischemic heart disease.^85,86^ Cord-blood cells can be induced into pluripotent stem cells for treating malfunctional mice retina,^87^ re-differentiated iPSCs are employed to cure brain lesions in mice with their motor abilities regained after the therapy.^88^

iPSCs are successfully used for organ regeneration, for example, ex vivo cardiomyocytes can be used to regenerate fetal hearts to normal hearts via the Yamanaka’s method.^89^ Human “liver buds” can be generated from three different cells including iPSCs, endothelial stem cells and mesenchymal stem cells.^90^ The bio-mimicking processes made the liver buds self-packaging into a complex organ for transplanting into rodents. It functions well for metabolizing drugs.^91^

Some iPSC applications are advanced to clinical stages. For example, a group in Osaka University made “myocardial sheets” from iPSCs, transplanted them into patients with severe heart failure, the clinical research plan was approved in Japan,^92^ patients are under recruiting. Additionally, two men in China received iPSC-differentiated cardiomyocytes treatments.^93^ They were reported to be in good condition although no detailed data are revealed.^93^ iPSCs derived from skin cells from six patients are reprogrammed to retinal epithelial cells (RPCs) to replace degenerated RPCs in an ongoing phase I clinical trial.^94^ Similarly, phase I clinical trials are also undergoing for thalassemia treatment using autologous iPSCs differentiated hematopoietic stem cells,^95^ patients are recruiting. Till now, no Phase III study on stem cell-related therapy has been conducted. The major concern is the safety of iPSCs with the carcinogenic possibilities: teratoma has been observed in iPSCs injected mice,^96^ low-induction efficiency, incomplete reprogramming of genomes, immunogenicity and vector genomic integrations are also issues of concerns.^97,98^ More efforts are required for clinical applications.

#### Synthetic biology in tissue engineering

Tissue engineering aims to repair damaged tissues and restoring their normal functions. The use of synthetic biology in tissue engineering allows control of cell behaviors. Artificial genetic constructs can regulate cell functions by rewiring cellular signals. As engineered cells are building blocks in tissues with special properties to achieve smarter functions, synthetic biology allows complex tissue engineering for new medical studies.

By overexpression of functional genes or transcriptional factors, stem cells can differentiate to generate specific tissue cells successfully.^99^ This is a simple and common way in stem cell-based tissue engineering. However, the gene overexpression lacks feedback control mechanisms to avoid excess nutrient consumption or cell toxicity.^100^ For an instance, constitutive overexpression of the anti-apoptotic factor Bcl-2 leads to tumorigenesis risks.^101,102^ CRISPR/dCas9 bioswitches or synthetic mRNAs are found able to solve the problem via time and spatial-specific expression of genes.^103,104^ Moreover, introductions of genetic circuits sensing small molecules or cell-surface proteins are well studied, especially Tet repressor-based system.^105^ Gersbach et al. designed a Tet-off system controlling Runx2 factors that can regulate the in vivo osteogenic processes.^106^ Yao et al. employed a Tet-on system to express Sox9 specifically in engineered rat chondrocytes, Sox9 is a key factor maintaining chondrocyte viability, activating the protein expressions for type II collagen and aggrecan in cartilage tissue engineering.^107^ Chondrocyte degradation was inhibited after Dox (Tet system inducer) injection in implanted cell scaffolds.^107^ The Tet-on system is also used for overexpressing interleukin-1 receptor antagonist (IL-1Ra) gene to modulate inflammatory cytokines during the chondrogenesis processes in cartilage repairs^108^ (Table 2). Tet-switches have aided elapsed time controllable gene expressions for tissue engineering.

**Table 2:** Synthetic biology in mammalian cell-based therapies

	Main features	Cell host /cell type	Genetic manipulations	Applications	Stages	Reference
Tisagenlecleucel (Kymriah)	CD19-targeted CAR-T cancer immunotherapy	Patient’s own T-cells	The chimeric antigen receptor (CAR) is composed of a murine single‐chain antibody fragment that recognizes CD19, fused to intracellular signaling domains from 4‐1BB and CD3‐ζ.	Acute lymphoblastic leukemia and diffuse large B‐cell lymphoma	Approved	^417^
Axicabtagene ciloleucel (Yescarta)	CD19-targeted CAR-T cancer immunotherapy	Patient’s own T-cells	Expressing a CAR comprising an anti‐CD19 single chain variable fragment linked to CD28 and CD3‐ζ costimulatory domains.	Diffuse large B‐cell lymphoma	Approved	^418^
Idecabtagene vicleucel (Abecma)	B-cell maturation antigen (BCMA)-directed CAR-T cell therapy	Patient’s own T-cells	Comprises an anti-BCMA single-chain variable fragment (scFv) fused to a CD8 linker region, the 4-1BB co-stimulatory and the CD3‐ζ signaling domains	Relapsed and refractory multiple myeloma	Approved	^419^
SynNotch	An engineered Notch receptor to construct Multi-antigen prime-and-kill recognition circuits to induce effective proteins	Patient’s own T-cells	A synNotch receptor that recognizes EGFRvIII or MOG, induces expression of a CAR.	glioblastoma	Pre-Clinical	^56,420^
HEK-β cells	Engineering a synthetic circuit into human cells that can sense the glucose concentration and to correct blood sugar concentration	HEK-293 cells	Ectopic expression of a calcium channel, expression of insulin under control of elements of the calcium-responsive NFAT promoter, expression of both a short version of the glucagon-like peptide (GLP-1), a known insulin secretagogue, and its receptor (GLP1R).	Diabetes mellitus	Pre-Clinical	^70,421^
Caffeine-stimulated advanced Regulators (C-STAR) system	Sensing caffeine to produce peptides for treating diabetes	HEK-293T or hMSC-hTERT cells	Overexpression of T2D-treating peptide shGLP-1 under STAT3 promoter	Type 2 diabetes	Pre-Clinical	^422^
Guanabenz-controlled genetic circuits	Guanabenz activates the secretion of peptides GLP-1 and leptin.	Hana3A cells	The cTAAR1 signal transduction is rewired to dose-dependently control expression of the glucagon-like peptide 1 (GLP-1) and leptin via an IgG-Fc linker under the induction of guanabenz.	The metabolic syndrome	Pre-Clinical	^423^
Green tea-triggered genetic control system	Engineering cells to respond to protocatechuic acid (PCA), a metabolite in green tea to treat diabetes in mouse and nonhuman primate models.	HEK-293 cells	Using PCA-ON sensor (artificial KRAB-PcaV fusion repressor) to overproduce insulin and shGLP-1	Diabetes mellitus	Pre-Clinical	^424^
Synthetic optogenetic transcription device	Light-controlled expression of the shGLP-1 peptide to attenuate glycemic excursions in type II diabetic mice	HEK-293 cells	Overexpression of melanopsin; P_NFAT_-driven expression of shGLP-1	Type 2 diabetic mice	Pre-Clinical	^425^
Red/far-red light-mediated and miniaturized Δphytochrome A (ΔPhyA)-based photoswitch (REDMAP) system	Small and highly sensitive light-inducible switch in mammalian cells	HEK-293 cells	The PhyA interaction domain FHY1 is fused to the VP64 to create a light-dependent transactivator (FHY1-VP64), the DNA-binding domain Gal4 is fused to phytochrome ΔPhyA to create a fusion light sensor domain (ΔPhyA-Gal4), the transactivator can bind to its synthetic promoter P_5 × UAS_ to initiate transgene expression, following exposure to far-red light (730 nm), the transactivator terminates transgene expression.	Type 1 diabetic (T1D) mice and rats	Pre-Clinical	^387^
Gene expression by radiowave heating	Heating of iron oxide nanoparticles by radiowaves remotely activated insulin gene expression in cultured cells or mouse models	HEK-293T cells	Heated iron oxide nanoparticles activate TRPV1 channel to pump calcium, the insulin gene is driven by a Ca^2+^-sensitive promoter.	Lowers blood glucose in mice	Pre-Clinical	^192^
Electronic control of designer mammalian cells	Using wireless-powered electrical stimulation of cells to trigger the release of insulin	Human β cells	Coupling ectopic expression of the L-type voltage-gated channel Ca_V_1.2 and the rectifying potassium channel K_ir_2.1 to the desired output through endogenous calcium signaling, the insulin gene is overexpressed by the system.	Type 1 diabetic mice	Pre-Clinical	^421^
Self-sufficient control of urate homeostasis	Senses uric acids levels and triggers dose-dependent derepression of a urate oxidase that eliminates uric acid	HeLa cells	The HucR start codon was modified (GTG→ATG) and fused to a Kozak consensus sequence for maximum expression, fusing it to the C terminus of the KRAB protein, eight tandem hucO modules downstream of the P_SV40_ promoter to drive the signal peptide-uricase cassette SS_Igk_-mUox.	Acute hyperuricemia in mice	Pre-Clinical	^426^
Dopamine sensors for hypertension control	A synthetic dopamine-sensitive transcription controller to produce the atrial natriuretic peptide to reduce blood pressure under pleasure situations	HEK-293 cells	Rewiring the human dopamine receptor D1 (DRD1) via cAMP to synthetic promoters containing cAMP response element-binding protein 1(CREB1)-specific cAMP-responsive operator modules to express atrial natriuretic peptide	Hypertension in mice	Pre-Clinical	^427^
Insulin self-regulation circuit for correcting insulin resistance	A self-adjusting synthetic gene circuit to reverse insulin resistance in diabetes and obesity animal models	HEK-293 cells	Ectopically express the human insulin receptor (IR) via the rewiring of the MAPK pathway, expression of adiponectin transgene (consisting of three tandem adiponectin molecules fused to a human IgG-Fc fragment), regulated by a synthetic promoter specific to the hybrid transcription factor TetR-ELK1	Insulin resistance in mice	Pre-Clinical	^428^
Smartphone-controlled optogenetically engineered cells	Remotely control release of glucose-decreasing proteins by engineered mammalian cells implanted diabetic mice under the control of far-red light	HEK-293 cells	The bacterial light-activated c-di-GMP synthase BphS and the c-di-GMP-specific phosphodiesterase YhjH are designed to regulate drug production according to user-defined glycemic thresholds.	Diabetes mellitus in mice	Pre-Clinical	^186^
Modified rapamycin-induced CAR-T cells	Engineered T cell “on” or “off” by administering small molecule rapalog	K562 cells	The part I constructs of the ON-switch are similar to conventional CAR, in addition to the FKBP domain for heterodimerization; part II variants contained the additional DAP10 ectodomain for homodimerization and the CD8α transmembrane domain for membrane anchoring.	Xenografted matched cancer cells	Pre-Clinical	^429^
Synthetic RNA regulatory systems for T-cell proliferation	Linking rationally designed circuit to growth cytokine targets to control mouse and primary human T-cell proliferation	CTLL-2 and T_CM_ cells	Using tetracycline-responsive switches to inactivate ribozyme and cell-proliferation associated cytokines that are expressed to promote T-cell growth.	N.A.	N.A.	^430^
Nonimmune cell cancer therapies	A new class of synthetic T-cell receptor-like signal-transduction device to kill target cells	HEK-293T and hMSC cells	Employs JAK-STAT signaling mediated by the IL4 and IL13 receptor, with STAT6 as a signaling scaffold, and uses CD45-mimetic molecule upon specific cell contact as an OFF/ON switching mechanism.	N.A.	Pre-Clinical	^431^
PD-1 and CTLA-4 based inhibitory chimeric antigen receptors (iCARs)	Designed antigen-specific inhibitory receptors to block these unwanted “on-target, off-tumor” responses.	Mice’s own T-cells	The cytoplasmic domain of CTLA-4 or PD-1 was fused to the human prostate-specific membrane antigen (PSMA) transmembrane domain.	Leukemia in mice	Pre-Clinical	^432^
Resveratrol-triggered regulation devices in CAR-T cells	Allow precise control over T cell activity through adjustment of resveratrol dosage	Mice’s own T-cells	RES_rep_ device consists of a resveratrol-dependent transactivator ResA_3_ that fused to a synthetic activator VPR via the C terminus of TtgR, the chimeric transactivator can bind to the resveratrol-dependent promotor P_ResA1_, positioned in front of a promoter P_hCMVmin_.	Mouse tumor model of B cell leukemia	Pre-Clinical	^433^
CAR-transduced natural killer cells (CAR-NT) in CD19-positive lymphoid tumors	CD19-targeted CAR-NK cancer immunotherapy	HLA-mismatched anti-CD19 CAR-NK cells	NK cells were transduced with a retroviral vector expressing genes that encode anti-CD19 CAR, IL-15, and inducible caspase 9 as a safety switch.	Non-Hodgkin’s lymphoma or chronic lymphocytic leukemia	Phase 1 and 2 trial	^434^
CAR-macrophages (CAR-M) for solid cancer immunotherapy	CD19-targeted CAR-M cancer immunotherapy	Human macrophage THP-1 cells	First-generation anti-CD19 CAR encoding the CD3ζ intracellular domain, targeting the solid tumor antigens mesothelin or HER2	Mice bearing SKOV3 lung or peritoneal metastases	Phase 1	^435^
In vivo gene editing to treat Duchenne muscular dystrophy (DMD)	CRISPR-Cas9 can correct disease-causing mutations in dog models of DMD.	Systemic delivery in skeletal muscle or vein	Using *Streptococcus pyogenes* Cas9 coupled with a sgRNA to target a region adjacent to the exon 51 splice acceptor site to correct the skipping of exon 51	Duchenne muscular dystrophy in dog	Phase 1 and 2 trial	^436^
In vivo gene editing to treat Leber congenital amaurosis 10 (EDIT-101)	Targeted genomic deletion using the CRISPR/Cas9 system for the treatment of mice and cynomolgus monkeys with LCA10 bearing the CEP290 splice mutation	Subretinal delivered in mice	A combination of specific pairs of sgRNAs and Cas9 to excise the intronic fragment containing the IVS26 splice mutation in *CEP290* gene	Leber congenital amaurosis 10 in mice or cynomolgus monkeys	Phase 1 and 2 trial	^437^
CRISPR-edited stem cells to treat human diseases	Transplanted CRISPR-edited CCR5-ablated HSPCs into a patient for acute lymphoblastic leukemia with HIV-1 infection	Edited CD34+ cells	Cells were transfected with a ribonucleoprotein complex comprising Cas9 protein and two designed guiding RNAs targeting CCR5.	Acute lymphoblastic leukemia patient with HIV-1 infection	Clinical	^438^
Mammalian synthetic cellular recorders integrating biological events (mSCRIBE)	Recording of molecular events into mammalian cellular genomic DNA	HEK-293T cells	This device consists of a self-targeting guide RNA (stgRNA) that repeatedly directs *Streptococcus pyogenes* Cas9 nuclease activity toward the DNA that encodes the RNA, when cellular sensors regulate the Cas9 activity, the device enabling localized, continuous DNA mutagenesis as a function of stgRNA expression.	N.A.	N.A.	^439^
Synthetic gene network for thyroid hormone homeostasis for Graves’ disease	A gene circuit that monitor increased thyroid hormone levels and drive the expression of a validated TSH receptor antagonist.	CHO-K1 cells	This synthetic control device consists of a synthetic thyroid-sensing receptor (TSR), a yeast Gal4 protein/human thyroid receptor-α fusion, which reversibly triggers expression of the TSH_Antag_ gene from TSR-dependent promoters.	Graves’ disease in mouse models	Pre-Clinical	^440^
Aroma-triggered pain relief based on synthetic cell engineering	Spearmint (R-carvone) induced analgesic peptide production in mice	Hana 3A cells	Ectopic expression of the R-carvone-responsive olfactory receptor OR1A1 rewired via an artificial G-protein deflector to induce the expression of a secretion-engineered and stabilized huwentoxin-IV variant	Relief chronic pain in mice	Pre-Clinical	^441^
Synthetic gene circuit controls human IPSCs differentiation	Using vanillic acid as the inducer for cell-fate gene expressions in the transition of hIPSCs to beta-like cells	hIPSC cells	vanillic acid-triggered expression switches for the transcription factors Ngn3 and Pdx1 with the concomitant induction of MafA	N.A.	Pre-Clinical	^442^
Cytokine-induced anti-inflammatory factors to treat experimental psoriasis	Designed and engineered human cells that sequentially detected elevated TNF and IL22 levels from a psoriatic flare and produced therapeutic doses of IL4 and IL10.	HEK-293T cells	Rewired TNFR-signaling through NF-κB to a synthetic NF-κB-responsive promoter that controlled the expression of human IL22 receptor α which enables IL22-mediated activation of the JAK signal transducer and activator of transcription STAT signaling cascade, driving expression of the cytokines IL4 and IL10.	Psoriasis in mice	Pre-Clinical	^443^
Designer exosomes to deliver therapeutic cargo into brain (EXOtic devices)	The device enhances exosome biogenesis, packaging of specific RNAs into exosomes, secretion of exosomes, targeting, and delivery of mRNA into the cytosol of target cells to treat Parkinson’s disease.	HEK-293T cells	Overexpressing STEAP3, SDC4, NadB and Cx43 variant S368A, targeting CHRNA7 receptor	Parkinson’s disease in mice	Pre-Clinical	^444^
Genetic-code-expanded cell-based therapy for treating diabetes in mice	A genetic code expansion-based therapeutic system, to achieve fast therapeutic protein expression in response to cognate ncAAs at the translational level	HEK-293T cells	A ncAA-triggered therapeutic switch (NATS) system composed of a bacterial aaRS-tRNA pair and an insulin gene carrying an ectopic amber codon	Diabetes in mice	Pre-Clinical	^445^
Synthetic mammalian cell-based microbial-control device	Detects microbial chemotactic formyl peptides through a formyl peptide sensor (FPS) and responds by releasing AI-2 to inhibit pathogens	HEK-293T cells	A FPS module that detects formylated peptides by FPR1, the adapter protein Gα16 redirects receptor signaling to the Ca^2^^+^ transduction pathway, constitutively expressed 5′-methylthioadenosine nucleosidase MTAN cleaving endogenous SAH and the LuxS under the control of a Ca^2^^+^-responsive promoter to produce AI-2.	Inhibit *Vibrio harveyi* and *Candida albicans*	Pre-Clinical	^446^
Human liver buds	Self-packaging into a complex organ using three stem cells	iPSCs, endothelial stem cells and mesenchymal stem cells	N.A.	Generation of a functional human organ from pluripotent stem cells	Pre-Clinical	^90^
Reprogramming of cardiomyocytes drives heart regeneration	Uses of ex vitro cardiomyocytes for regeneration of fetal hearts to normal hearts by the Yamanaka’s method	Patient’s own cardiomyocyte cells	Cardiomyocytes specific expression of OSKM (Oct4, Sox2, Klf4, and c-Myc) is enabled by administration of doxycycline.	Heart failure in mice	Pre-Clinical	^89^

*N.A.* not applicable

The optogenetic induction systems are also used in the control of cell behaviors in tissue engineering. Light inducible proteins are able to respond to UV and far-infrared lights, making light induction applicable.^109^ Various optogenetic circuits are constructed by fusing light-sensitive motifs to well-characterized transcriptional factors.^110,111^ Spatial-specific gene activation has been successfully employed to guide the arrangement of cells.^112^ Sakar et al. used blue light-induced channel rhodopsin-2 to achieve dynamic and region-specific contractions of tissues.^113^ The optogenetic control of engineered murine-derived muscle cells offers remote gene activation or silencing via the light-sensitive membrane Na^+^ channel and ion-inducible downstream elements for tissue engineering.

Inspired by successes of CAR-T cells, G protein-coupled receptors (GPCRs) are engineered to sense artificial ligands for tissue engineering.^114^ Park et al. successfully designed and used a GPCR sensing clozapine-N-oxide (CNO) in primary cells for the control of cell migration in response to CNO concentration gradients.^115^ This technology could make a valuable module for wound healing and cell regeneration. Synthetic biology makes possible to program cells to multicellular structures in a self-assembly manner.^116^ Toda et al. employed synNotch methods to engineer cell adhesion signals in a population of mouse fibroblasts that were turned into multilayers and polarized according to the synNotch receptor types.^117^

Besides cells, biomaterials are commonly used in tissue engineering, served as scaffolds and bio-mimicked organs.^118^ the auto-modulation characteristics of biomaterials in response to stimuli or chemical compounds are useful in biomaterial-based tissue engineering. Baraniak et al. engineered the B16 cell line with a green fluorescent protein (GFP) reporter induced by RheoSwitch Ligand 1 (RSL1), which was coated on poly(ester urethane) films, allowing GFP activation for up to 300 days on the film.^119^ Deans et al. constructed an isopropyl-β-d-thiogalactoside (IPTG)-induced Lac-off system in Chinese hamster ovary (CHO) cells, and IPTG encapsulated in poly(lactide-*co*-glycolide) (PLGA) scaffolds or PEG beads was released in a sustainable manner. The reporter gene indicated that the induction lasted over 10 days in mouse models implanted subcutaneously into the dorsal region,^120^ the GFP fluorescence level was observed to be controlled by its locations.^121^ The spatial-induced gene expression regulation has become a design-of-concept in many applications like cartilage repair and in vivo 3D cell scaffolds.

In summary, expressions of biological circuits could generate functionalized cells for tissue engineering. Multiple synthetic biology designs *e.g*. time and spatial-dependent gene expression, induction and autoregulation systems and smart biomaterials are available in this field. The state-of-the-art development still remains with many obstacles from moving truly synthetic tissues into clinic, but at least some foundations are settled for future studies.

### Engineered bacterial cells for therapeutical applications

Synthetic biology approaches have promoted genetically engineered bacteria for novel live therapeutics (Fig. 2).^122^ Bacteria containing synthetic gene circuits can control the timing, localization and dosages of bacterial therapeutic activities sensing specific disease biomarkers and thus develop a powerful new method against diseases.^123^ Synthetic biology-based engineering methods allow to program living bacterial cells with unique therapeutic functions, offering flexibility, sustainability and predictability, providing novel designs and toolkits to conventional therapies.^124^ Here some advances are presented for engineered bacterial cells harboring gene circuits capable of sensing and transduction of signals derived from intracellular or extracellular biomarkers, also the treatments and diagnosis based on these signaling pathways. The concept of bacterial cell-based live therapeutics and diagnostics are rapidly growing strategies with promises for effective treatments of a wide variety of human diseases.

#### Engineered bacterial cells in cancer diagnosis and treatments

Some anaerobic/facultative anaerobic bacterial cells are good candidates for tumor treatments. They can target the anaerobic microenvironment of tumors, they also have the tumor lysis-inducing and trigger inflammation abilities useful in fighting against solid tumors.^125^ Engineered microbes can become suitable tools for cancer in vivo diagnosis. Danino et al. engineered *E. coli* with LacZ reporter gene, the bacterium produces LacZ when in contact with tumor cells. Subsequently, mice were injected with chemiluminescence substrates for LacZ (Table 3). The luminescence is enriched in the urine to generate red color.^126^ The method is more sensitive than microscopes as it can detect tumors smaller than 1 cm. Similarly, Royo et al. constructed a salicylic acid-induced circuit converting 5-fluorocytosine to toxic products in attenuated *Salmonella enterica* for tumor killing.^127^ *Salmonella enterica* localized in tumor tissues after the injection, with the additional providing of salicylic acid (inducer) and 5-fluorocytosine (substrate), tumor cells were eliminated via the formation of 5-fluorouracil from the bacterial cells.

**Table 3:** Synthetic biology in microbe-based therapies

	Main features	Microorganism type	Genetic manipulations	Applications	Stages	Reference
SYNB1020	Transform ammonia into L-arginine to treat hyperammonia	*E. coli* Nissle 1917	Deleted the gene *argR*, *thyA* and integrated the gene *argA215*, under the control of the *fnrS* promoter (P_*fnrS*_)	Hyperammonia in mice and cynomolgus monkeys	Phase 1b/2a	^150^
SYNB1618	Engineered *Escherichia coli* Nissle to express genes encoding Phe-metabolizing enzymes to treat phenylketonuria	*E. coli* Nissle 1917	Two chromosomally integrated copies of *pheP* and three copies of *stlA* under the regulatory control of P_*fnrS*_, two additional copies of *stlA* were placed under the control of the P_tac_ promoter.	Phenylketonuria	Phase 1/2	^151^
Probiotic-associated therapeutic curli hybrids (PATCH)	Genetically engineer *Escherichia coli* Nissle 1917 (EcN) to create fibrous matrices that promote gut epithelial integrity in situ	*E. coli* Nissle 1917	CsgA fused to TFF3, under the control of an inducible promoter (P_BAD_)	Dextran sodium sulfate (DSS)-induced colitis in mice	Pre-Clinical	^145^
Engineered bacteria for colorectal-cancer chemoprevention	The engineered *Escherichia coli* bound specifically to colorectal cancer cells and secreted myrosinase to transform small molecule form broccoli to anticancer agents.	*E. coli* Nissle 1917	Expressed and secreted YebF-I1 myrosinase catalyzes the glucosinolate hydrolysis to sulforaphane, while the expression of INP-HlpA facilitates bacterial CRC cell binding.	Colorectal-cancer in mice	Pre-Clinical	^447^
Bacteria engineered to reduce ethanol-induced liver disease	Bacteria engineered to produce IL-22 induce expression of REG3G to reduce ethanol-induced steatohepatitis	*Lactobacillus reuteri*	*L. reuteri* EF-Tu promoter drives murine IL-22 gene.	Ethanol-induced liver disease in mice	Pre-Clinical	^154^
Bacteria-mediated tumor therapy triggered via photothermal nanoparticles	Bacteria are coated with nanogold particles (or indocyanine green-loaded nanoparticles) able to receive light for heat generation, inducing therapeutic protein TNF-α in tumor sites.	*E. coli* MG1655/ attenuated *Salmonella typhimurium*	A widely used temperature-sensitive plasmid pBV220 containing TcI repression and tandem pR-pL operator-promoter was introduced to express human TNF-α.	Breast tumor in mice	Pre-Clinical	^448,449^
Engineered bacteria overexpressing anti-inflammatory cytokines	Therapeutic dose of IL-10 can be reduced by localized delivery of a bacterium genetically engineered to secrete the cytokine.	*Lactococcus lactis* MG1363	lactococcal P1 promoter driving *usp45* secretion leader fused to the *mIL-10* gene.	Murine colitis	Pre-Clinical	^450^
Modified bacteria producing peptides to inhibit obesity	Engineered bacteria that express the therapeutic factor N-acylphosphatidylethanolamines (NAPEs) into the gut microbiota	*E. coli* Nissle 1917	Overexpressing N-acyltransferase At1g78690 under the intrinsic promoter from *Arabidopsis thaliana*	Obesity in mice	Pre-Clinical	^149^
Synthetic genetic system to eliminate gut pathogens	A gene encoding an anti-biofilm enzyme induced by *P. aeruginosa*-specific quorum sensing signal	*E. coli* Nissle 1917	Genes *alr* and *dadX* are deleted, the 3OC_12_-HSL-inducible promoter drives the expression of DspB and E7.	*Pseudomonas aeruginosa* gut infection in *Caenorhabditis elegans* and mice	Pre-Clinical	^146^
Bacteria synchronized for drug delivery	Engineer a clinically relevant bacterium to lyse synchronously and routinely at a threshold population density to release genetically encoded cargo	attenuated *Salmonella enterica serovar* Typhimurium	The *luxI* promoter regulates production of autoinducer (AHL), which binds LuxR and enables it to transcriptionally activate the promoter, negative feedback arises from cell death that is triggered by a bacteriophage lysis gene (*φ*X174 E) which is also under control of the *luxI* promoter.	Subcutaneous liver metastasis in mice	Pre-Clinical	^451^
Engineering bacteria to serve as whole-cell diagnostic biosensors	Sensing abnormal glucose concentrations in human urine samples	*E. coli* DH5α	Combinatorial of pCpxP and pYeaR promoters driving expression of integrase TP901 and BxB1	Detection of urine glucose in human samples	Pre-Clinical	^135^
Engineered bacteria as live diagnostics of inflammation	Engineered a commensal murine *Escherichia coli* strain to detect tetrathionate, which is produced during inflammation.	*E. coli* strain NGF-1	*ttrR/S* genes and the P_ttrBCA_ promoter from *S. typhimurium* to drive Cro expression, and inserted it into the genome, containing the phage lambda cI/Cro genetic switch can sense and record environmental perturbations.	Detection of gut inflammations	Pre-Clinical	^452,453^
Recording of cellular events over time using engineered bacteria	A multiplexing strategy to simultaneously record the temporal availability of three metabolites (copper, trehalose, and fucose) in the environment of a cell population over time.	*E. coli* BL21	The *E. coli cas1-cas2* cassette is downstream of the P_LTetO-1_ promoter, the CopA/GalS or TreR sensors drive RepL.	Recording specific environmental factors surrounding the cells	Pre-Clinical	^454^
Ingestible micro-bio-electronic device (IMBED) for in situ biomolecular detection	Heme-sensitive probiotic biosensors demonstrate accurate diagnosis of gastrointestinal bleeding in swine via miniaturized luminescence readout electronics that wirelessly communicate with an external device.	*E. coli* Nissle 1917	The heme biosensor P_L(HrtO)_, overexpressing HrtO and ChuA, *luxCDABE* was used as the output of the genetic circuit to generate luminescence captured by electronic devices.	Gastrointestinal bleeding in swine	Pre-Clinical	^455^
Probiotics detect and suppress cholera	Engineered an *L. lactis* strain that specifically detects quorum-sensing signals of *V. cholerae* in the gut and triggers expression of an enzymatic reporter that is readily detected in fecal samples.	*L. lactis* subsp. cremoris MG1363	Designed an *L. lactis* hybrid receptor that combines the transmembrane ligand binding domain of CqsS with the signal transduction domain of NisK, placed the gene *tetR* downstream of the chimeric repressor-controlled *nisA* promoter to enable constitutive repression of an engineered *Bacillus subtilis xylA-tetO* promoter.	Detection of cholera infection in mice	Pre-Clinical	^456^
Engineering probiotics for detection of cancer in urine	Orally administered diagnostic that can noninvasively indicate the presence of liver metastasis by producing easily detectable signals in urine.	*E. coli* Nissle 1917	Genomic expression of *luxCDABE*, IPTG-inducible *lacZ* in plasmid	Indicate the presence of liver metastasis	Pre-Clinical	^126^
Underwater adhesives made by bacterial self-assembling multi-protein nanofibers	Fusing mussel foot proteins of *Mytilus galloprovincialis* with CsgA proteins	*E. coli* C3016 strain	Overexpression of the fused protein in *E. coli*	Novel bio-adhesive	N.A.	^248,250^
Engineered modularized receptors activated via ligand-induced dimerization (EMeRALD) to detect pathological biomarkers	Build EMeRALD receptor detecting bile salts in *E. coli* by rewiring bile salt-sensing modules from *Vibrio cholerae* and *Vibrio parahaemolyticus*	*E. coli* strain NEB10β	Synthetic bile acid sensor TcpP-TcpH for taurocholic acid; synthetic bile acid sensor VtrA-VtrC for taurodeoxycholic acid, driving sfGFP as the reporter	Detection of bile acid concentration in serum	Pre-Clinical	^457^
Kill tumor cells via salicylic acid-induced circuit	*Salmonella* spp., carrying an expression module encoding the 5-fluorocytosine-converting enzyme cytosine deaminase in the bacterial chromosome or in a plasmid, to mice with tumors	attenuated *Salmonella enterica*	Carries an expression module with a gene of interest (cytosine deaminase) under control of the XylS2-dependent Pm promoter	Eliminate xenografted tumor in mice	Pre-Clinical	^127^
Ultrasound-controllable engineered bacteria for cancer immunotherapy	Engineer therapeutic bacteria to be controlled by focused ultrasound to release of immune checkpoint inhibitors	*E. coli* Nissle 1917	TcI42-containing thermal switch to express αCTLA-4 and αPD-L1 nanobodies in high temperature generated from the ultrasound	Eliminate xenografted tumor in mice	Pre-Clinical	^458^
Living bacterial polymer materials in gastrointestinal tract	Auto-lysis bacteria contain self-assembly materials to glue microbes up for stabilizing gut microbiota.	*E. coli* MC4100Z1	Cells carrying the ePop circuit produce ELPs fused with either multiple SpyCatcher or SpyTag sequences	Maintain gut microbes under perturbations by antibiotics	Pre-Clinical	^459^
Ketone-producing probiotics as a colitis treatment	Develop a sustainable approach to treat chronic colitis using engineered EcN that can sustainably release 3-hydroxybutyrate	*E. coli* Nissle 1917	The *ldhA* gene is knocked-out, *phaB*, *phaA* and *tesB* genes are overexpressed under *fnrS* promoter in the genome.	Acute colitis in mice	Pre-Clinical	^147^
Optotheranostic nanosystem for ulcerative colitis via engineered bacteria	Developed diagnosis and treatment kits containing two parts: the optical diagnosis sensor to smartphone processing and (ii) treatment based on optogenetic probiotics	*E. coli* Nissle 1917	A light-responsive EcN strain containing light-inducing promoter pDawn to drive *mil-10* gene for IL-10 production	Ulcerative colitis in mice	Pre-Clinical	^389^
SYNB1891	Targets STING-activation to phagocytic antigen-presenting cells (APCs) in the tumor and activates complementary innate immune pathways.	*E. coli* Nissle 1917	The CDA-producing enzyme DacA from *Listeria monocytogenes* was expressed in EcN under P_fnrS_ promoter, both *dapA* and *thyA* deleted in the genome.	Murine melanoma tumors and A20 B cell lymphoma tumors	Phase 1	^460^
Bacterial flagellin triggered enhanced cancer immunotherapy	Engineered a *Salmonella typhimurium* producing the flagellin B protein from another bacterium *Vibrio vulnificus* to induce an effective antitumor immune response	*S. typhimurium*	*relA* and *spoT* genes were deleted in the genome, the *pelB* leader sequence was fused to the upstream of *flaB* to guide extracellular secretion, under the control of a P_BAD_ promoter.	Mice colon tumors	Pre-Clinical	^461^
Quorum-sensing *Salmonella* spatial-selectively trigger protein expression within tumors	Integrated *Salmonella* with a quorum-sensing (QS) switch that only initiates drug expression in the tightly packed colonies present within tumors	attenuated *Salmonella enterica*	The p*luxI* promoter controls one operon consisting of genes encoding for proteins LuxR, GFP, and LuxI, LuxI produces the communication molecule 3OC6HSL.	Controlled therapy for mammary cancer in mice	Pre-Clinical	^462^
Tumor-specific lysis and releasing anti-cancer agents	Engineered a non-pathogenic *Escherichia coli* strain to specifically lyse within the tumor microenvironment and release an encoded nanobody antagonist of CD47	*E. coli* Pir1^+^	A stabilized plasmid that drives constitutive expression of a hemagglutinin (HA)-tagged variant of CD47nb, the strain overexpresses *luxI* and lyses at a critical threshold owing to the production of ϕX174E, resulting in bacterial death and therapeutic release.	Eliminating planted melanoma, mammary tumor in mice	Pre-Clinical	^463^
Engineered probiotics for regularly self-lysis to release nanobodies	Engineered a probiotic bacteria system to release nanobodies targeting the immune checkpoints	*E. coli* Nissle 1917	The PD-L1nb and CTLA-4nb sequences were cloned onto separate plasmids downstream of a strong constitutive *tac* promoter on a high-copy plasmid, an HA protein tag was added to the 3′ end of the nanobody sequences.	Colorectal cancer and B cell lymphoma in mice	Pre-Clinical	^464^
Engineering of symbiont bacteria in mosquitos to control malaria	*Serratia* AS1 was genetically engineered for secretion of anti-*Plasmodium* effector proteins, and the recombinant strains inhibit development of *Plasmodium falciparum* in mosquitoes.	*Serratia* AS1	The five effector genes were cloned in a single construct, (*MP2*)_2_-*scorpine*-(*EPIP*)_4_-*Shiva1*-(*SM2*)_2_, under the control of a single promoter.	Malaria prevention	N.A.	^465^
Engineered bacterial communication prevents *Vibrio cholerae* virulence	*Escherichia coli* Nissle 1917 to express the auto inducer molecule cholera autoinducer 1(CAI-1) to increase the mice’s survival in cholera infections	*E. coli* Nissle 1917	Express the gene *cqsA*, under control of the native constitutive promoter P_fliC_	Prevents *Vibrio cholerae* virulence	Pre-Clinical	^466^
Noninvasive assessment of gut function: Record-seq	A CRISPR-based recording method (Record-seq) to capture the transcriptional changes that occur in *Escherichia coli* bacteria as they pass through the intestines	*E. coli* MG1655	An anhydrotetracycline (aTc)-inducible transcriptional recording plasmid consisted of FsRT-Cas1–Cas2 and CRISPR arrays	N.A.	N.A.	^467^

*N.A.* not applicable

To improve the effects of bacteria-based cancer therapies, some studies aim to further enhance bacterial tumor tropism.^128^ Some bacteria have natural affinity for the anaerobic environment of solid tumors, like *E. coli* or attenuated *Vibrio cholerae*, *Salmonella typhimurium*, and *Listeria monocytogenes*.^128^ However, the affinity is not sufficient for targeted therapies, bacterial cells in vivo are still dispersed in general. They can be augmented by introducing synthetic surface adhesins targeted to bind cancer-specific molecules like neoantigens or other chemicals or proteins that are enriched in cancer cells, not accumulated in somatic cells. Engineering of adhesins are demonstrated to be effective in enhancing bacterial tumor reactions. The adhesins are membrane-displayed proteins with extracellular immunoglobulin domains that can be engineered via library directed evolution screens. Piñero-Lambea et al. constructed a constitutive genetic circuit in *E. coli* with an artificial adhesin targeting green fluorescent protein (GFP) as the evidence of a proof of concept, it demonstrated the abilities from that binding of the cell membrane-engineered bacteria to GFP-expressing HeLa cells are successful both in vitro and in mice.^129^ Importantly, the intravenous delivery of this engineered bacteria to mice resulted in effective and efficient colonization in xenografted solid tumors of HeLa cells at a dose 100 times lower than that for a bacterial strain expressing an irrelevant control adhesin, or for the wild-type strain, suggesting that similarly engineered bacteria can be used to carry therapeutic agents to tumors at low doses with marginal potential systemic basal toxicities.^130,131^ However, few tumor-targeting bacteria have entered clinical stages. The facultative anaerobe *Salmonella typhimurium* VNP2000, has been engineered for safety with anti-tumor abilities in pre-clinical studies,^132^ yet it failed in the phase I clinical trial for marginal anti-tumor effects and dose-dependent side effects.^133^ Some other clinical investigations based on bacteria *Clostridia novyi*-NT or *Bifidobacterium longum* APS001F are ongoing or recruited for their phase I trials.^134^

#### Engineered bacterial cells for diabetes diagnosis and treatments

Bacteria have been engineered to detect glucose concentrations for diabetes. Courbet et al. described an approach in sensing abnormal glucose concentrations in human urine samples.^135^ They encapsulated the bacterial sensors in hydrogel beads, glucose in urine will change the color to red in beads. The in vitro bacterial glucometer has found outperforming the detection limit of urinary dipsticks by one order of magnitude.

Some proteins and peptides are biosynthesized in engineered gut bacteria for diabetes treatments. The engineered probiotic *L. gasseri* ATCC 33323 produced GLP-1 protein, the bacterium is orally delivered to diabetic rats,^136^ demonstrating a down-regulation of blood glucose levels to 33%. Similarly, engineered *L. lactis* FI5876 was reconstructed to biosynthesize and deliver incretin hormone GLP-1 to stimulate β-cell insulin secretion under conditions of high glucose concentrations. Results showed the glucose tolerance is improved in high-fat diet mice.^137^ The probiotic *L. paracasei* ATCC 27092 is engineered to secret angiotensin (1-7) [Ang-(1-7)], increasing the concentrations of Ang-(1-7) (an anti-inflammatory, vasodilator and angiogenic peptide phamarceutical), and reduced the side effects on retina and kidney in diabetic mice, as the insulin production level is increased after oral administration of the bacteria. Following the design, oral uptake of engineered *B. longum* HB15 which produces penetratin (a cell-penetrating peptide with the ability of enhancing delivery of insulin), and GLP-1 fusion protein also enhanced the production of GLP-1 in the colorectal tract.^138–140^ *L. paracasei* BL23 was also successfully designed to produce monomer GLP-1 analogs displayed to the bacterial membrane via fusing GLP-1 to peptidoglycan-anchor protein PrtP, the engineered bacteria enhanced glycemic control in rats with diabetes. However, the efficacy is still limited and needed further investigations.^141^ In addition to GLP-1, some other proteins like the immunomodulatory cytokine IL-10 along with human proinsulin were simultaneously introduced to engineered *L. lactis* MG1363, the combination therapy with low-dose systemic anti-CD3 allowing reversal of irregulated self-autoimmune triggered diabetes in non-obese diabetic mice.^142,143^ This design could possibly be effective for the treating of type 1 diabetes in human.

#### Engineered bacterial cells for diagnosis and treatments of gastrointestinal diseases

Probiotics can be used to treat inflammatory bowel disease (IBD).^144^ IBD is chronic inflammation of tissues in the digestive tract, including ulcerative colitis and Crohn’s disease. Patients are suffering from diarrhea, pain and weight loss. Synthetic biology approaches and ideas help bacteria acquire more powerful abilities against gastrointestinal diseases. Praveschotinunt et al. designed an engineered *E. coli* Nissle 1917 (EcN) that produces extracellular fibrous matrices to enhance gut mucosal healing abilities for alleviating IBD in mice.^145^ Curli fibrous proteins (CsgA) were fused with trefoil factor (TFF) domains to promote the reconstruction of cell surface, and the bacterium could produce fibrous matrices via the in situ protein self-assembly of the modified curli fibers. The results revealed that the designed EcN significantly inhibited the production of pro-inflammatory cytokines, alleviated the weight loss of mice, maintained colon length, demonstrating its anti-inflammation ability in the dextran sodium sulfate (DSS)-induced acute colitis mouse model. The design could be expanded to a general approach for probiotic-based live therapeutics in IBD treatments.

Bacteria are feasible to be engineered to directly eliminate pathogens for preventing infectious diseases in gastrointestinal tracts. *Pseudomonas aeruginosa* is a common multidrug-resistant pathogen difficult to treat. Engineered EcN has been employed for the detection, prevention and treatment of gut infections by *P. aeruginosa*.^146^ The designed EcN was able to sense the biomarker N-acyl homoserine lactone produced by *P. aeruginosa*, and autolyzed to release a biofilm degradation enzyme dispersin and pyocin S5 bacteriocin to eliminate the pathogen in the intestine. Moreover, the reprogrammed bacteria displayed long-term (over 15 days) prophylactic abilities against *P. aeruginosa* and was demonstrated to be more useful than treating a pre-established infection in mouse models. 3-Hydroxybutyrate (3HB) is a component of human ketone bodies with therapeutic effects in colitis. Yan et al. constructed an EcN overexpressing 3HB biosynthesis pathway.^147^ Compared to wild-type EcN, the engineered *E. coli* demonstrated better effects on mouse weights, colon lengths, occult blood levels, gut tissue myeloperoxidase activity and proinflammatory cytokine concentrations.^147^ However, the studies are the preliminary results in mice, they have not reached clinical trials yet. Further efforts are needed to evaluate their applications in human.

#### Engineered bacterial cells for metabolic disorders

Engineered gut microbes also have been used to target metabolic disorders.^148^ *E. coli* was designed to treat obesity synthesizing anorexigenic lipids precursors in mice with high-fat diet.^149^ Some efforts are made to degrade toxic compounds accumulated in patients via live bacteria. Kurtz et al. engineered an *E. coli* Nissle 1917 strain for converting ammonia to L-arginine in the intestine and reducing systemic hyperammonemia in both mouse and monkey models.^150^ Isabella et al. reprogrammed *E. coli* Nissle 1917 to overexpress phenylalanine degradation pathway to metabolize excess phenylalanine in phenylketonuria (PKU) patients. In the Pah^enu2/enu2^ PKU mouse model, oral uptake of the engineered bacterium significantly down-regulated blood phenylalanine concentration by 38%.^151^

Alcoholic liver disease is the major cause of liver disorders, widely risking the health of heavy drinkers.^152^ The engineered *Bacillus subtilis* and *L. lactis* could be employed to express ethanol degradation pathway (alcohol dehydrogenase and aldehyde dehydrogenase) for the detoxification of alcohol and alleviate liver injury from alcohol overconsumption.^153^ Moreover, the lectin regenerating islet-derived 3 gamma (REG3G) protein is decreased in the gastrointestinal tract during chronic ethanol uptake. *L. reuteri* was designed to overexpress the interleukin-22 (IL-22) gene, which increased REG3G abundance in the intestine, reduced inflammation and damage in liver using an alcoholic liver disease mouse model.^154^

Synthetic biology approaches have allowed the construction and design of engineered live biotherapeutics. Many cases are targeting future clinical applications. The examples discussed here indicate that, with the development of circuit designs and understanding in microorganism hosts, researchers can construct live biotherapeutics that function in a precise, systematic, inducible and robust manner. However, many efforts are still needed to weaken bacterial toxicity and increase the controllability in vivo.

## Synthetic biology in the fabrication of emerging therapeutic materials

**Fig. 4:**
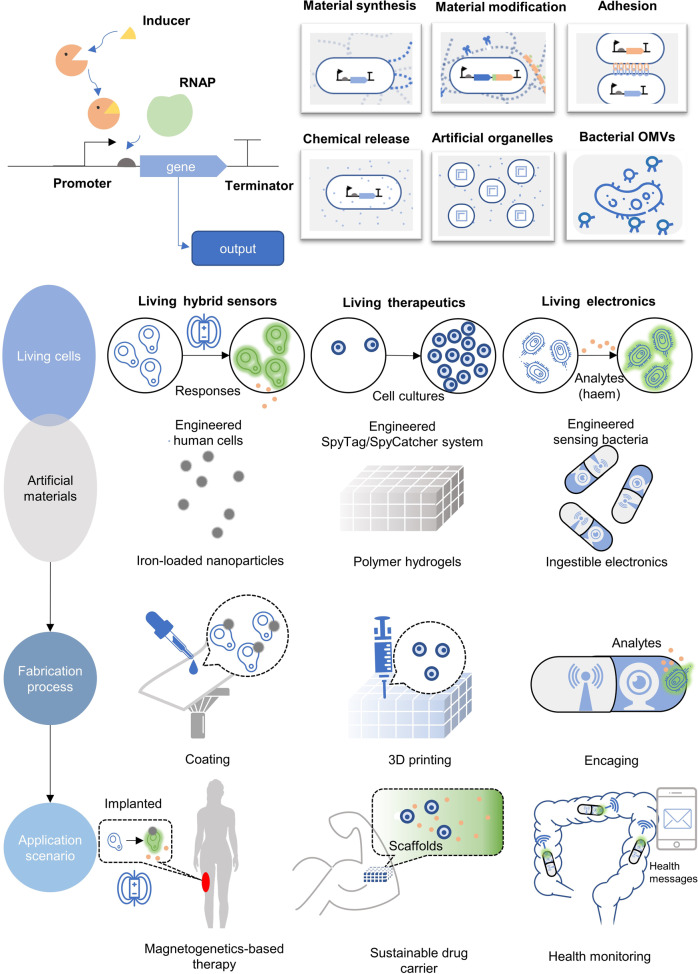
The designs and applications in synthetic material biology. Generally, a genetic circuit is constructed to synthesize biological materials or sense environments. The engineered bacteria are endowed with new characteristics like color change and unique surface properties. The applications for cells with excellular matrices are diverse including magnet field induced therapies, development of novel drug carrier or health monitoring via sophiscated biofabrication processes. This figure is partially inspired by the paper^469^

Besides engineered cells, engineered nanomaterials are also commonly used in medical fields. Nanobiotechnology aims to solve important biological concerns similar to drug delivery, disease diagnosis and treatment based on its unique physical, chemical and biological properties of micro-nano scale materials^155,156^ (Fig. 4). Nanomaterials possess unique mechanical, magnetic and electronic properties, able to respond to external signals, controlling their downstream circuits.^157^ However, traditional nanomaterials are generated from physical and chemical processes, the solvents and modifying molecules are frequently causing bio-safety issues.^158^ Recently, biological nanomaterials have been developed exhibiting their advantages in environmentally friendly, enhanced biocompatibility and bioactivity, and low tissue toxicity under the guidance of synthetic biology.^159^ Based on synthetic biology concepts and approaches, the genetic engineered bacteria,^160^ yeast^161^ and tobacco mosaic virus^162^ (TMV) can serve as bio-factories for nanomaterials.^163^ Mammalian cell-derived vesicles and nanoparticles have suitable biocompatibility, also commonly used as nanomedicines.^164^ Biological materials can be constructed and engineered with the help of synthetic biology, extending their application scenarios in modern disease treatments.

### Synthetic biology in the artificial organelles

Following the principles of synthetic biology, biocatalysis or trigger-sensing modulus nanoparticles can be processed to self-assembly organelles,^165,166^ which are biomimicry of characteristics of living cells like enzyme reaction compartmentalization and stimuli-responses (Fig. 4). The design also provides new inputs for constructing artificial cells.^167^ Additionally, combinations of artificial organelles and engineered living cell chassis including CAR-T cells and engineered bacteria, the nano-living hybrid system can exert its dual effects to enhance therapeutic results or more strictly control of artificial systems.

Polymersomes are artificial hollow vesicles made by amphiphilic polymers, using as shells of artificial organelles. van Oppen et al. employed a polymersome-based system that was anchored with cell-penetrating peptides on its outer membrane. The artificial organelles possess inside catalase, allowing degradation of external reactive oxidative molecules, perform as a synthetic organelle, protecting the cells from ROS damages triggered via H_2_O_2_, which showed abilities in uptaking by human primary fibroblasts and human embryonic kidney cells.^168^ A similar design relying on polymersomes equipped with two enzymes and related transmembrane channels, was used to mimic cell peroxisomes. These organelles were able to deal with both H_2_O_2_ and superoxide radicals. The results further demonstrated the feasibility of artificial organelle with catalase activity. Based on similar ideas, engineered polymersomes may play a role in treating medical conditions including Parkinson’s, Alzheimer’s, Huntington’s, metabolic diseases, cancers and acatalasemia via harboring various therapeutic proteins inside of the artificial organelles.^169,170^

Moreover, the fusion of nanobiotechnology and synthetic biology may achieve novel functions. First, researchers can create “artificial lives” via assembling nanoparticles following the “bottle-up” principle. The idea can be applied in constructing biological components using inorganic scaffolds and functional nanomaterials with nucleic acids and protein inside of the nanoparticles.^171,172^ The “top-down” principle, or engineering natural cells for actual demands, can be used as a guidance when using nanomaterials in living cells for chimeric biological systems to increase the robustness, stability and sensitivity in specific medical applications.

### Constructing nanoparticle-mediated genetic circuits

Auto-responses can be achieved via internal environmental stimulus to induce genetic switch ON/OFF^173^ (Fig. 4). However, the irreversible situation of genetic switches is a common and difficult problem.^174,175^ To circumvent the weakness of genetic constructs, nanoparticles are employed to sense signals for the transductions in vivo. Light, sound, heat and magnet stimuli are easy to respond for nanoparticles, they can be used as inducer systems for solid tumor and diabetes treatments. Yet the spatial-specific induction is hard for physical stimulus.^176^ Overall, via combining the advantages of genetic sensor and nanoparticles, it is feasible to convert physical stimuli into genetic switch with specified input signals by introducing nanoparticles for signal transduction, and the time-spatial control of gene expressions are realized.^177^

Near-infrared (NIR) light-responsible gene circuits are feasible for in vivo therapeutical applications for their better transmission of NIR light able to penetrate tissues and lower toxicity.^178^ NIR-sensing protein is identified in plants and bacteria, like the bacterial phytochromes (BphPs).^179^ However, NIR-sensing proteins are generally with low brightness.^180^ Also, the lacking of structural information hindered their rational engineering.^180^ To circumvent the disadvantages of NIR light-responsible protein, researchers have use nanomaterials converting NIR light into visible light. For example, Chen et al. employed nanoparticles doped with lanthanide to derive 980 nm NIR light into visible light, controlling genetic gates of opsin-expressing neurons in mice models.^181,182^ Another design uses plasmonic gold nanorods or photothermal responsible nanoparticles to transduct NIR light into up-regulation of temperature, then the promoters of heat-shock protein are activated for downstream gene expression.^183,184^ One disadvantage for nanoparticles is that they must be injected into human body, it could be solved by developing genetically engineered nanoparticles.^185^ Similar to magnetogenetics, in which biosynthesized ferritin can be used as a tool to prepare exogenous paramagnetic nanoparticles. However, the penetration depth needs much improvements in these samples (less than 1 cm), which is not enough for the applications of cell therapy demands in humans. Some researchers couple light-generating microdevices with photosensitive engineered therapeutic cells to address the problem (Fig. 4),^186–188^ patients can control the release of drugs via applications of their own smartphone or real-time monitoring their health. Besides, some genetic-encoded luminescent module can produce light in situ with a protein like various luciferases, all emit the desired wavelength with corresponding substrates. The in vivo light induces the photosensitive proteins that trigger transgene expressions for customized demands.^189^

In addition to optogenetics, magnetogenetics emerges for regulating the cell activities and has been applied for controlling of nanomaterial therapies remotely and non-invasively (Fig. 4).^190–193^ Magnetic fields can penetrate human body without losses, which is a preferred characteristic in deep-tissue targeted therapies. Previous magnetogenetics tools are mainly externally injected magnetic nanoparticles.^190,192,194^ The nanoparticles are usually with radius of <10 nm, toxicity free and water-soluble.^190^ Heating of nanoparticles using remote magnetic fields can activate temperature-sensitive cation channels in cells. the next-generation tools are heterologously expressed receptor-targeted ferritin proteins in the form of nanoparticles (iron-loaded particles) in engineered cells, which could sense and transduce magnetic signals to cell membrane-anchored receptors like transient receptor potential channel 1 (TRPV1) or TRPV4.^191,193^ The membrane receptors are ion channels allowing calcium influx with the magnet stimuli. The described gene circuit can be manipulated to control NFAT-dependent transcriptional regulators for downstream functional genes. Implanted engineered therapeutic cells can achieve target-specific treatments and precise control of therapeutic dosage, time and location under magnetic fields.

However, the mechanisms of the magnetic activation of the sensor channels are still not clear, the theories proposed are under debate for a long time.^195^ TRPV channels are activated by a variety of signals including but not limited to mechanical forces and heat. Recently, a new mechanism is raised to solve the problem that how radio-frequency weak magnetic fields (1 mT) could trigger transient responses in living cells with ferritin-anchored TRPV channels.^196^ The mechanism is the dissociation of free Fe^3+^ from ferritin protein, resulting in an enhanced oxidation of membrane lipids via increased production of reactive oxygen species (ROS).^196^ These oxidized lipids have the ability to turn on the TRPV channels, resulting in calcium influx.^196–198^ Recently, ROS is reported to be involved in the treatment of combined electric and static magnetic fields in type 2 diabetic mice to increase their insulin sensitivity.^199^ In this research, low-energy fields can induce the expression of nuclear factor erythroid 2-related factor 2 (Nrf2), a transcriptional regulator controlling ROS levels.^199^ Moreover, the local ROS accumulation does not have side effects in mice, it is promising to induce gene expression via electromagnetic fields mediated by redox states.^200^ Magnetogenetics are exhibiting its potentials in remote control and targeted therapies. However, more efforts are needed to establish the magnetogenetic platform. Despite improvements in recent years, the cell toxicity and biocompatibility are two main obstacles of magnetic nanoparticles that still challenges their in vivo applications.

### Synthetic biology in drug delivery

The synthetic biology constructs are usually encapsulated in carriers for their functions in vivo. The safety concerns of viral vectors restrict their applications for editing human genome.^201^ Therefore, non-viral carriers are attracting more and more attentions. Nanotechnology can aid to deliver therapeutic agents including genetic circuits and genome engineering tools.^202,203^ With the advances in nanotechnology, more choices are available for targeted and controllable-release in DNA/RNA delivery system.^204^

One of the examples, the DNA/RNA delivery system based on liposome nanomaterials, has become an effective and potential gene therapy method, with a variety of artificial lipid vectors approved for clinical uses. For example, an RNAi therapeutic agent under the trade name Onpattro, has been developed by Alnylam Pharmaceuticals. The drug was approved in 2018 for the treatment of polyneuropathy.^205^ Liposomes are small lipid vesicles, the size is between 50 nm and 1 μm.^206^ Liposome are generally amphiphilic consisted with a hydrophobic tail and a hydrophilic head, employed for delivering drugs in various treatments.^207^ Because liposomes reduce drug toxicity, deliver drugs directly to targets via site-specific injections, and envelope drugs free from degradation, they have advantages over traditional drug therapies in delivery. CRISPR/Cas9-aided gene therapies are commonly using lipid-based nanoparticles integrating negatively charged mRNA, gRNA scaffolds and CRISPR genes with positively charged liposomes via electrostatic interactions.^208^ Felgner et al. first designed and used liposomes by enveloping DNA and delivered it to target mammalian cells in the plasma membrane, leading to DNA expression after its endocytosis.^209^ The liposome vector not only helps therapeutic DNAs to pass through the cell membrane barrier, but also protects them from DNase degradation and immune responses to maintain their activities. Partially inspired by the results that liposomes can be applied in human therapies, liposomes also have delivered mRNA encoding SARS-CoV-2 antigens to humans as vaccines. Both the Moderna mRNA-1273 and BioNTech/Pfizer BNT162b2 vaccines are encapsulated in liposomes, with their clinical use approvals.^210^

Nanotechnology can also aid synthetic biology to deliver chemicals.^211,212^ Nanocarriers deliver chemicals minimize off-target effects,^213,214^ enhancing therapeutic results^215,216^ compared to traditional drug administrations. External physical stimuli can also initiate the release of chemicals to make the system sustainable and controllable.^217^ Here, we discuss the application of synthetic biology-guided biological chemical carriers.

The genetically encoded post-translational modified protein can self-assemble to carry hydrophobic drugs.^218^ The protein with different structure and material properties can be easily manipulated at the amino sequence level. Based on synthetic biology approaches, Mozhdehi et al. designed and co-expressed an elastin-like polypeptide and an N-myristoyl transferase in *E. coli*.^219^ The N-myristoyl transferase enzyme modified the polypeptide with myristoyl groups in bacteria, generating a temperature-induced self-assembly behavior.^219^ The lipid core of the purified recombinant protein can carry hydrophobic compounds with a prolonged drug half-life.^220^ The protein can form complex assembly systems encapsulated with chemicals. Li et al. used an in silico designed cationic chimera near-infrared fluorescent protein and anionic carboxylate-terminated PEG to prepare a protein-PEG nanocarrier.^221^ The nanoprotein is amphiphilic, resulting in the aggregation and phase separation in aqueous solutions to form nanoparticles.^221^ The engineered nanoparticle achieved imaging of solid tumor and metastasis in vivo without transfections for the fluorescent nature of the protein,^221^ as well as the nanoprotein served as the long-term drug carrier, which can improve half-life and therapeutic effects of IL1-Ra significantly.^222^

### Engineered bacterial outer-membrane vesicles (OMVs) as nanocarriers

Bacterial outer membrane vesicles (OMVs) are lipid spheres released from Gram-negative bacterial outer membranes, they can be used for trafficking biochemicals to other cells in the environment.^223^ The gene manipulation methods from synthetic biology can improve bio-originated nanoparticle abilities,^224^ expanding the application scenarios of outer-membrane vesicles (OMV) and engineered cells.^225,226^

Engineered OMV anchored with recombinant proteins are potentially used in medical and clinical fields (Fig. 4). The general strategy to surface display proteins in the engineering of OMV is to fuse their genes together in the OMV expression system. Many studies have employed the *E. coli* Cytolysin A (ClyA) protein as the fusion chassis to anchor exogenous proteins to OMV membranes.^227–230^ In recent studies, ClyA has been reported to successfully fuse to the domain 4 of *Bacillus anthracis* protective antigen, to extracellular domain of the influenza A matrix protein 2 (M2), and to GFP without influences OMV formation.^231^ The alternative strategy is to express proteins to the periplasm and assembly to the OMV when the fusion step hampers protein functions.^232^ However, the heterologous protein is enveloped inside of the OMV, which is a main disadvantage of the strategy. Bartolini et al. also employed the method to carry *Chlamydia muridarum* protein HtrA in OMVs as a vaccine against *Chlamydia* infections.^233,234^ Some proteins from *Streptococcus* spp. are expressed to the periplasm with the *E. coli* OmpA signal peptide to packed them into OMVs.^235^ Even though these proteins are located inside of the OMV, they were able to activate the immune responses,^232,233,235^ the generated IgG antibodies had strong activity to specific pathogens in murine models.^225,232,235^ The results indicated that antigen location is not a decisive factor in OMV-elicited immune responses.

Besides proteins, OMVs can be engineered to carry chemicals. LPS and capsular polysaccharides (CPS) decorating the cell membrane of pathogens are also vaccine candidates.^236^ However, polysaccharides trigger immune responses apart from T-cells, the immunological memory cannot be established.^237^ To circumvent the problem, polysaccharides are anchored to nanocarriers to elicit immunological memories. Polysaccharide and capsule synthesis genes are expressed in *E. coli*, packed into OMVs using the mentioned methods. The designed OMVs are potentially used as vaccines after further optimizations. Chen et al. employed the O-antigen polysaccharide from *Francisella tularensis*, the genes were heterologous expressed in *E. coli* to produce the glyco-modified OMVs.^238,239^ Mice injected with the engineered OMVs were protected against *F. tularensis* strains.^238^ Another similar design uses *Streptococcus pneumoniae* CPS (Sp-CPS) biosynthesis genes. They were overexpressed in *E. coli*, located both on the membrane of engineered OMVs and bacterial cells.^240,241^ After the vaccination via injecting these collected OMVs, the vaccine was effective in opsonophagocytosis assays and IgG antibodies were triggered against Sp-CPS.^240^ In general, synthetic biology approaches have developed better engineered OMVs for immunotherapies,^242,243^ with bright prospects in drug targeted-delivery and combined therapies.

### Biomimetic medical adhesive materials

Traditional medical adhesive materials are limited in underwater uses, which hampered their applications in body fluids. Recently, some biomimetic designs are conducted to solve the problem based on synthetic biology ideas (Fig. 4).^244^ Many marine organisms (e.g. mussel and barnacle) have extraordinary adhesive capacities to rock surfaces,^245,246^ as they produce L-3,4-dihydroxyphenylalanine (DOPA) as an important component of the adhesion proteins in underwater surfaces.^247^ Zhong et al. reported a strong underwater adhesive by fusion of CsgA curli protein and mussel foot proteins.^248^ The excellent design reconciled the biocompatibility and adhesion activity, with the prospect of in vivo applications like tissue repairs. Zhang et al. is inspired by natural biomaterials like bones and mussel foots,^249^ they developed a *Bacillus* spp. extracellular matrix-based living glue.^250^ The live material is adhesive with regeneration abilities. Engineered mammalian cells could be constructed with adhesive proteins, serving as in vivo live functional glues. As summarized above, the novel live biomedical adhesives are hotspots in medical synthetic biology. However, most studies are focused in the material properties rather than their biocompatibility and biodegradability, adequate efforts are needed to promote the material for clinical applications.

### Genetically encoded click chemistry in medical applications

Inspired by click chemistry, isopeptide bond was engineered for the establishment of protein-protein linkages.^251^ The genetic-encoded click chemistry is more applicable in living organisms compared with traditional click chemistry. The SpyTag/SpyCatcher system is an application of the natural click-like reaction among Gram-positive bacterial pilus,^252,253^ using biological ways to form stable chemical bonds between amino acids, additional modifications of biomacromolecules are not needed in click chemistry-oriented proteins (Fig. 4).^254^ Genetically encoded click chemistry (or Spy chemistry) is a powerful tool for materials made via synthetic biology.^255^

Hydrogels are cross-linked hydrophilic polymer networks,^256^ serving as carriers for biomacromolecules and stem cells due to their biocompatibilities and extracellular matrix (ECM) like properties.^257^ Hydrogel materials synthesized using chemical polymerizations are facing bioactivity problems.^258^ The protein characteristics are decided by amino acid sequences. Protein hydrogels are easier to synthesize and be controlled using various DNA sequences. Yang et al. employs the SpyTag/SpyCatcher system to synthesize a 4-arm star-like light-sensing protein. The protein can form rapid sol-gel and gel-sol phase transitions in response to AdoB_12_ and light, respectively.^259^ Biofilm-degrading glycosyl hydrolase PslG can be enveloped into the hydrogel, endowing the material with abilities against multidrug-resistant bacteria in chronic infections. Sun et al. designed a Spy-network containing multiple SpyTags and SpyCatchers in elastin-like proteins and the leukemia inhibitory factor. The proteins were turned into a high-mechanical strength hydrogel, allowing mouse embryonic stem cells to maintain pluri-potentials without adding other cytokines in the gel.^260^

Genetically encoded click chemistry has also used in the vaccine development. Some designed proteins can self-assembly into virus-like particles (VLPs) to surface display antigens for mimicking pathogens.^261^ Synthetic vaccines are causing more and more attentions for their efficiency and safety compared to canonical vaccines developed from dead or attenuated microorganisms. Genetically encoded click chemistry is a useful approach to modify the surface with heterologous antigens to enhance their immunogenicity.^262,263^ The easy formation of chemical bonds based on Spy chemistry provide a customized and convenient method to design synthetic vaccines via encoded protein self-assembly. Liu et al. developed a synthetic vaccine using the SpyCatcher/SpyTag chemistry via covalently ligating specific antigens and chemicals. The result demonstrates this engineered vaccine targets dendritic cells successfully.^264^ The generated protein-chemical hybrid vaccine remained the individual functions and had the ability to trigger B and T cell responses. Brune et al. engineered virus-like particles (VLPs) via exhibiting SpyCatcher on material surfaces, further enabling the modification of VLPs with SpyTag-expressing malarial antigens to develop novel vaccines.^265^ The VLP-antigen vaccine can trigger immune responses rapidly and efficiently via only one single immunization, indicating the potential of this effective, simple, and modular modification method.

### Genetic code expansion for medical and pharmaceutical applications

A protein usually consists of 20 natural amino acids. To add non-canonical amino acids (ncAAs) into proteins, the genetic code expansion technology has been developed.^266^ ncAAs can be used to modify proteins via conjugation with peptides or chemicals depending on actual demands. Employing a termination codon (UAG/UGA/UAA), the heterologous bioorthogonal aminoacyl-tRNA synthase (aaRS)-tRNA pairs can add ncAAs to any site in a protein.^267^ Many different aaRS/tRNA pairs have been developed.^268–270^ The high-efficiency genetic code expansion devices allow the production of ncAA-containing protein and multiple ncAA-inserted proteins.^271,272^ The ncAA insertions are succeed in all main model organisms.^273,274^ Applications of the genetic code expansion system in medical fields are summarized here.

#### Genetic code expansion for antibody-drug conjugates

The antibody-drug conjugates (ADC) combine antigen-recognizing abilities of antibodies and tumor-killing capacities of chemicals commonly used in tumor therapies.^275^ Traditional ADC drugs are chemical modification of cysteines or lysines in the antibodies, which may affect the immunogenicity, stability and half-life.^276^ With the development of genetic code expansion technology, the introduction of a functional ncAA in the antibodies are feasible.^277^ The site-specific, high-efficiency conjugation between antibodies and chemicals can be achieved. Oller-Salvia et al. developed a novel genetic code expansion system incorporating a cyclopropene derivative of lysine into antibodies.^278^ The antibody conjugates to monomethyl auristatin E (MMAE) via a rapid Diels-Alder reaction.^278^ The resulting ADC was stable and effective in serum. Wang et al. conjugated the Lck inhibitor dasatinib to monoclonal antibody CXCR4 using genetic code expansion methods.^279^ The ADC avoids the side reactions during the chemical modification. The resulting dasatinib-antibody conjugate inhibited T-cell activation with low EC_50_ with negligible effects on cell viability.

#### Genetic code expansion in the bispecific antibodies

Bispecific antibodies (BsAb) possess two specific antigen binding sites with enhanced tumor-killing abilities.^280^ Some BsAbs have been approved by FDA.^281^ The traditional BsAb production method relies on fusions of proteins, resulting in steric hindrance in the ligand-binding domains.^282^ Additionally, the antibody production is at a low level with short half-life.^283^ Synthesis of BsAbs via chemical modifications meets similar questions to ADC productions.^284^ Genetic code expansion methods can conjugate two antibodies via a PEG linker to circumvent the challenges. Kim et al. introduced a ncAA (pAcF) to the antigen-binding fragment Fab region of anti-HER2 and anti-CD3 antibodies to form BsAb via two-step reactions.^285^ Picomolar concentrations of the BsAb induced effector-cell mediated cytotoxicity in vitro. Employing the Diels-Alder reaction between tetrazine-containing ncAA and bicyclononyne- containing ncAA, a BsAb recognizing BCMA was developed to treat multiple myeloma,^286^ successfully overcoming the drug-resistances in patients with multiple myeloma.

#### Genetic code expansion for engineering adeno-associated viruses (AAV)

AAVs are small parvovirus infecting human and primates.^287^ AAVs are commonly used in gene therapies to achieve non-pathogenic, broad host range and high transfection and expression efficiencies.^288^ However, the controllability and targeting ability are limited, hampering their applications. Zhang et al. used genetic code expansio to enhance the targeting ability of AAVs, conjugating cyclic arginyl-glycyl-aspartic acid (cRGD) to the shell protein of AAVs for targeting integrin.^289^ Erickson et al. engineered AAVs for opto-control of the infection.^290^ The R585 and R588 residues in vp1 protein of AAV2 were replaced by a light-sensitive ncAA, which hampered the interaction of vp1 and HSPG protein, resulting in inhibiting the infection of AAV. Exposed to UV light would remove the light-sensing moiety, recovered the infecting abilities of AAVs.^290^ The method enhances time-spatial controllability of AAV vectors.

#### Genetic code expansion for prolonging a protein half-life

PEG is commonly used in prolonging the half-life of therapeutic proteins.^291^ However, the random-modified PEG usually influences binding sites of therapeutic agents.^292^ Thus, genetic code expansion may provide advantages in modifying proteins. Cho et al. used genetic code expansion to site-specifically modify PEG in human growth hormone, which is highly instable in clinical applications.^293^ The modified human growth hormone is also with good batch to batch repeatability during the manufacturing processes. Some ncAAs increase protein stabilities per se. Xuan et al. demonstrated incorporation of a reactive isothiocyanate group into proteins to improve the heat-stability of myoglobin. Stable thiourea crosslinks were formed between the proteins.^294^ Similar designs using long chain thiol-containing or fluorinated ncAAs were also verified.^295,296^

#### Genetic code expansion for developing novel vaccines

ncAAs provide a wide variety of modifications of potential antigens that are candidates for vaccines. Gauba et al. inserted ncAAs containing nitrophenyl moiety into murine TNF-α protein for strong antibody response even with adjuvants.^297^ ncAA-addicted genetically modified organism (GMO) is useful for vaccine developments.^298^ The inactivated or attenuated pathogen-based vaccines usually have reduced effectiveness.^299^ Construction of a GMO strain that relies on ncAA to survive has been conducted to amplify live-virus vaccines. By introducing a termination codon in the genome of influenza A virus, HIV-1 or hepatitis D virus, the viruses can only replicate in engineered cells with specific aaRS/tRNA pairs and ncAAs. Si et al. inserted a termination codon in the NP protein of influenza A viruses, leading to a stronger immunogenicity and triggering broader immune responses.^300^ Based on the same idea, more and more live bacterial vaccines are under development.^298^ However, bacteria are more complex compared to viruses. Many mutation mechanisms can help bacteria to escape from expression terminations.^301^ The termination escapes restrict further applications with genetic code expansion in bacteria. Mandell et al. constructed a bacterium that metabolically dependent on ncAAs for survival.^302^ The bacterium exhibited unprecedented resistance to evolutionary escapes, providing a hint to the development of live bacteria vaccines.

#### Other medical applications of genetic code expansion

The genetic code expansion technology can be applied for the construction of controllable CAR-T cells. Incorporation of p-azidophenylalanine (pAzF) into the Fab allows the identification and conjugation of fluorescein isothiocyanate (FITC), activating the antibody for cancer treatments.^303^ Changing the inducer FITC to a short peptide was also proven applicable in cancer therapies.^304^ FITC or peptides were used as inducers of CAR-T cells that provide a more safety-control approach for immunotherapies. The genetic code expansion has also been applied for biosynthesis of peptide natural products. Nisin is a complex lanthipeptide with broad-spectrum of anti-bacterial activities. Zambaldo et al. introduced a number of ncAAs into nisin, equipping it with novel macrocyclic topologies with enhanced activities.^305^

The genetic code expansion methods are developing rapidly, modifying proteins both in vivo and site-specifically. The most sophisticated organism for this method is zebrafish and mouse.^306^ The method should be improved to apply in more higher species. Although more than 200 different ncAAs have been used for genetic code expansion, most ncAAs are based on similar structural units. Enriching structure types is another direction for developments. In the future, genetic code expansion technology will bring more delicate treatments for mankinds.

## Synthetic biology in the biosynthesis of therapeutic drugs

**Fig. 5:**
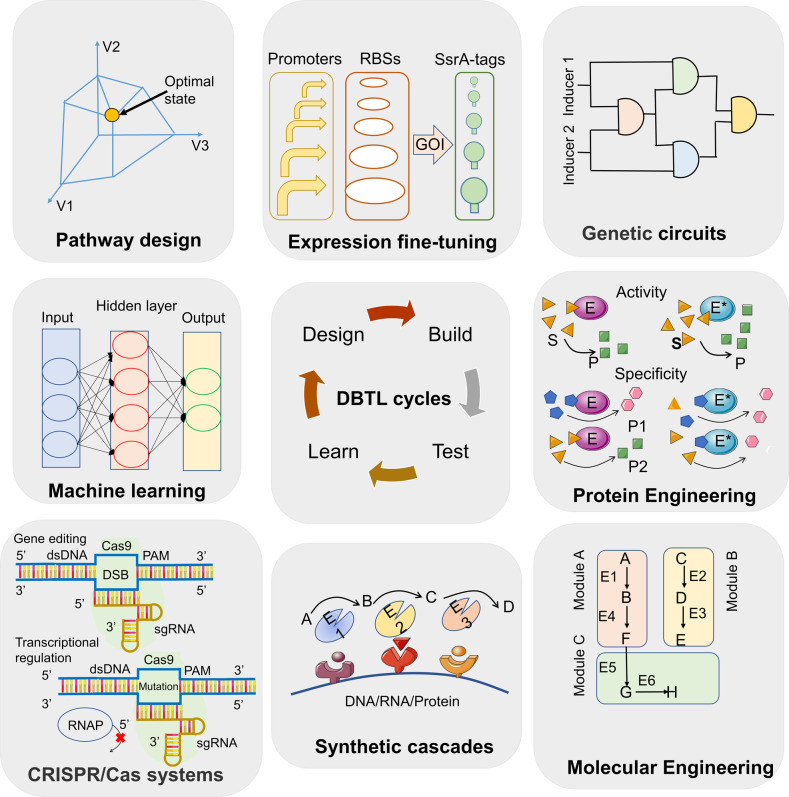
Technologies commonly used in synthetic biology. Various synthetic biology methods and tools have been developed to promote the design-build-test-learn cycle of cell factory construction, and these technologies are reforming the medical uses for synthetic biology. Pathway design is the first step, primary results are acquired via the constructed genetic circuits. Some optimizations are needed before next-round of tests, and the characteristics of the system is better understood from preliminary data. The design-build-test-learn cycles are iterative processes to improve robustness and efficacy of synthetic biology systems

In the recent years, synthetic biology approaches has become promising in sustainable and cost-effective production of phamarceuticals. Synthetic biology designs (Fig. 5) and constructs biological circuits or chassis including bacteria, yeasts, cell cultures or whole plants, for effectively producing high-value added phamarceutical products or phamarceutical intermediates. It offers a scalable and sustainable way for productions of bioproducts using CO_2_ based substrates, the production is rapid and robust, feasible for the large-scale industrial production, bioproducts can be manufactured without excessive cultivating and harvesting of medicinal plants (Table 1).

As a classical field in synthetic biology, synthesis of pharmarceuticals is different from other medical applications. it generally uses yeast or bacteria as the production chassis. Synthetic biology concepts are extensively used in microorganisms, especially the DBTL (design-build-test-learn) (Fig. 5). DBTL cycle comprises the molecular biology designs and constructs in the beginning, and the experimental results are the basis for the new cycles of designs. The single-cell systems are easier to be manipulated than mammalian cells, In manmalian systems, the DBTL cycle can take very long, which is also an obstacle for mammalian synthetic biology. In the microbial synthesis of drugs, high-throughput screening and directed evolution are commonly used to accelerate experimental paces. Synthetic biology in microbes points to the direction of manmalian synthetic biology in a sense.

### Biosynthesis of terpenoid drugs

Terpenoids are 5-carbon compound isoprene derivatives, also the largest group of plant secondary metabolites comprising approximately 60% of identified natural products.^307^ Many of them are bioactive medical ingradients.^308^ The anti-malaria drug, artemisinin, is sesquiterpene lactone containing an endoperoxide bridge.^309^ Initially, artemisinin was extracted from the plant *Artemisia annua*^310^ with a very low (0.01%-1%) content,^311^ much less than the actual medical demands. The chemical route to artemisinin is difficult and inefficient mainly due to the multiple-chiral centers of this molecule.^312^ The microbial synthesis of artemisinin prodrugs lowered drug cost. Biosynthesis of amorphadiene was a milestone in synthetic biology. The recombinant *E. coli* synthesized initially only 24 µg caryophyllene equivalent/ml.^9^ After continuous optimizations, another artemisinin prodrug, namely, artemisinic acid, reached 25 g/L produced by engineered yeast.^22,23^ The biosynthesis of artemisinic acid is a successful example of synthetic biology.

Taxol is a diterpene extracted from Pacific yew trees, serving as an anti-cancer agent.^313^ Its production mainly relies on laborious and low-efficiency plant cell cultures.^314^ Ajikumar et al. engineered *E. coli* cells to produce a taxol precursor, taxadiene, at a titer of 1 g/L.^315^

The ginsenosides are triterpene saponins found in the plant genus *Panax* with cancer prevention and anti-aging effects.^316^ Using the yeast cell-factory, various ginsenosides including ginsenoside Rh2 and ginsenoside compound K are synthesized with the titers of 2.2 g/L and 5.0 g/L, respectively.^317,318^ Microbial approach reduces the shortage of ginsenoside for clinical uses.

### Biosynthesis of alkaloid drugs

Alkaloids are a variety of organic compounds containing at least one nitrogen atom.^319^ As a natural product, alkaloids are commonly used as they have pharmacological activities.^320^ Biosynthesis of alkaloids circumvent the bans on growing certain plants like poppy and marijuana.^321^ The formation of chiral centers during biosynthesis also outcompetes chemical synthesis for most chiral alkaloid compounds.^322^ Galanie et al. employed engineered yeast cells to produce thebaine and hydrocodone.^323^ Overexpression of 21 genes (for thebaine) or 23 genes (for hydrocodone) led to their formations of 6.6 × 10^−5^ g/L and 3 × 10^−7^ g/L, respectively. Nakagawa et al. improved the process using *E. coli* chassis.^324^ The titers for thebaine and hydrocodone were enhanced to 2.1 × 10^−3^ and 4 × 10^−5^ g/L, respectively. The production of opiates reached miligram level. Subsequent metabolic engineering are needed to promote biosynthesized opiates to meet market demands.

Similar to the biosynthesis of artemisinic acid, cannabinoids are natural products from cannabis, commonly used for pain killing and anxiolytic actions.^325^ (S)-Tetrahydropalmatine and cannabigerolic acid are two well-known cannabinoid hard to extract from plants.^326^ The biosynthesis processes for cannabigerolic acid were established by Luo et al. The yield from yeast reached 0.1 g/L.^327^ (S)-Tetrahydropalmatine biosynthesized by yeast by Hafner et al. reached 3.6 × 10^−6^ g/L, a successful concept-of-proof for microbial production of complicated cannabinoids.^328^

### Biosynthesis of amino acid-derivative drugs

Using amino acids as building blocks, amino acid derivatives are also played an important role in human health.^329^ This class of compounds is usually synthesized via biological routes rather than chemical synthesis for their multiple chirality moieties. Compared with alkaloid and terpenoids, amino acid-derivatives are more simple in structures with diversity.^329^ Psilocybin is a L-tryptophan derivative with effects of anti-drug-addiction, relieving depression and anti-post-traumatic stress disorder effects.^330^ *E. coli* or *Saccharomyces cerevisiae* have been engineered to heterologously express the synthetic pathways, forming 1.2 g/L and 0.6 g/L psilocybin, respectively.^330,331^ Dencichine, also known as β-*N*-oxalyl-*L*-α,β-diaminopropionic acid (β-ODAP), is a plant metabolite first isolated from *Lathyrus sativus* seeds. Dencichine can induce platelet aggregation in human blood, and it is the main effective component of the Chinese medicine Yunnan Baiyao.^332,333^ The authors optimized metabolic flux to dencichine in *E. coli* to the production with final titer reaching 1.29 g L^−1^ and a yield of 0.28 g g^−1^ glycerol.^334^ Microbial production of dencichine exhibits an example of employing artificial enzymes and pathways to produce a desired chemical in synthetic biology applications.

### Biocatalytic of asymmetric synthesis

Synthetic biology can assist multiple chiral-center chemical developments. Sitagliptin (Januvia) is a commonly used diabetes treatment, inhibiting DPP-4 enzyme in a competitive manner, reducing the cleavage of GLP-1 to increase the secretion of insulin.^335^ The market of Januvia reached 1.4 billion dollars by 2021.^336^ For chemical synthesis of sitagliptin, the chiral amine is transferred via a rhodium-based chiral catalyst with a low stereoselectivity and the product contaminated with rhodium.^337^ A transaminase and synthetic-biology-based engineering approach based on homologous modeling and saturation mutagenesis, a process was developed that substantially improved the efficiency and purity for sitagliptin synthesis.^337^

## Cell-free synthetic biology in medical applications

**Fig. 6:**
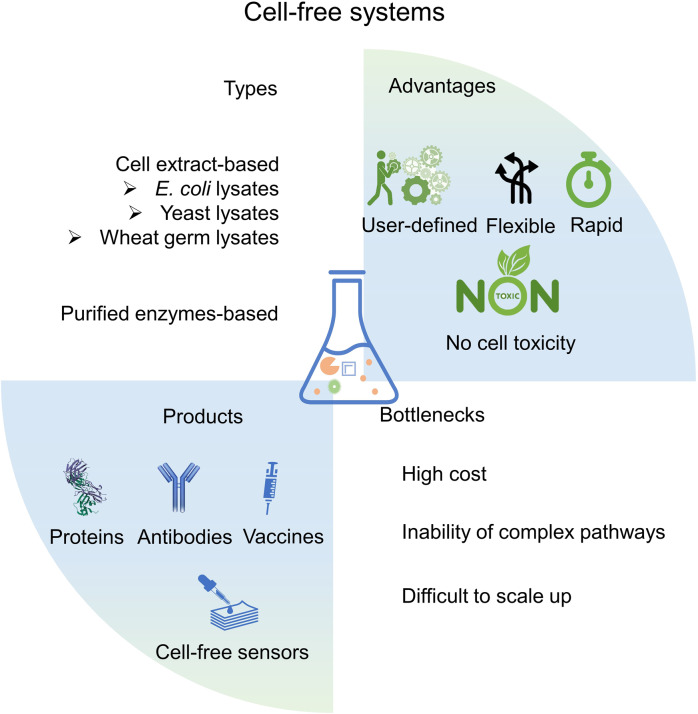
The charasteristics of cell-free synthetic biology. The types, advantages, products, and bottlenecks of cell-free systems are summarized in this figure. Generally, cell-free systems are used to produce pharmaceuticals or served as in vitro sensors. The main advantages are convenient, flexible and high tolerance to cytotoxicity. After solving the problems like high cost and instabilities, the system is promising for actual medical applications

Till now, efforts in synthetic biology have mainly focused on reprogramming organisms, development of genetic circuits and biological modules. However, because our knowledge on how life works is limited, the complex feature of creatures hindered progresses in synthetic biology. User-defined systems can solve the problem. Cell-free system is prepared to perform in vitro biological activities free from living cells (*e.g. tr*anscription and translation).^338^ As it is open, easy to control, flexible and high tolerance to cytotoxicity,^339,340^ the system has been used in synthesizing proteins that are difficult to express or toxic in cells (Fig. 6).^341^ Moreover, cell-free systems fit well to high-throughput screening.^342^ Recently, with the development of cell-free biosensing diagnosis^343^ and the advances in lyophilization,^344^ the applications of cell-free synthetic biology have expanded into medical and pharmaceutical fields.^345^

### Cell-free synthetic biology in pharmaceutical protein synthesis

Protein and peptide drugs are target-specific mostly with high activities and low toxicity for medical uses.^346–348^ Many well-known drugs are proteins or peptides like Trastuzumab (Herceptin),^349^ Adalimumab (Humira),^350^ Insulin Glargine (Lantus)^351^ and 13-valent pneumococcal conjugate vaccine (PCV13).^352^ 70% of the protein drugs are produced using the CHO cells.^353^ However, some proteins are toxic for growth of cell hosts.^354^ Cell-free protein synthesis (CFPS) provides a solution to the toxicity problems.^355^ Additionally, screening of intracellular proteins are feasible in CFPS systems,^356^ also lyophilization technologies allow the cell-free system to maintain highly active after one-year preservation.^357^

The cell-lysate based- and purified component systems are two commonly used CFPS systems.^358^ Theoretically, any organism could be used as the source in cell-lysate based system. The most common cell extract is from *E. coli*, wheat germ and yeast.^359^ *E. coli* lysate is frequently used for protein synthesis,^360^ wheat germ lysates for construction of protein arrays,^361,362^ yeast lysates for synthesis of glycoproteins.^363^ The purified component system comprises all purified translational-elements. Shimizu et al. developed a cell-free system using 36 transcription/translation related enzymes with highly purified ribosomes.^364^ The system is efficient although minimum. However, the high cost of purified components hampers its applications. The cell-lysate based system is the first choice of CFPS systems.

Vaccination is the most effective way for pandemic prevention.^365^ Cell-free systems provide a platform for rapid production of vaccines. Kanter et al. developed a cell-free system for highly effective production of a fusion protein consisting of a single chain Fv antibody fragment (scFv) connected to granulocyte-macrophage colony-stimulating factor (GM-CSF), a vaccine of B-cell lymphoma.^366^ Lu et al. described a CFPS overexpressing a domain of pandemic H1N1 influenza virus for potentially and broadly protective influenza vaccines.^367^ Besides bacterial systems, eukaryotic cell-free systems can express complex vaccines. Tsuboi et al. successfully expressed three malarial proteins in yeast lysate based cell-free systems, which is hard to produce in recombinant cells.^368^

Antibodies are important for disease treatments and diagnosis.^369^ CFPS is commonly used during the synthesis of antibodies. Ryabova et al. successfully produced functional scFv fragments in *E. coli* lysate-based cell-free system.^370^ Post-translational modification (PTM) is the final maturation step of proteins.^371^ Glycosylation is the main form of PTM important for maintaining the half-life and activity of protein drugs including some antibodies.^372,373^ CFPS can also introduce functional PTM to proteins. Jaroentomeechai et al. used CFPS to synthesize N-glycosylated scFv using *E. coli* cell-free systems.^374^ Overall, cell-free systems are useful complements to recombinant expressing systems for their rapid and on-demand properties.

### Cell-free synthetic biology for diagnosis

Generally, detection of pathogens are based-on biosensors.^375^ The sensing elements include enzymes, transcriptional factors, antibodies, organelles, whole-cells and tissues.^376–380^ Although many biosensors are rapid and sensitive, the disadvantages are including the instability of enzymes, biosafety concerns of whole-cell biosensors and the complexity in preparing microfluidic sensors.^293,381^ Therefore, cell-free sensors are developed. Pellinen et al. used luciferase as the reporter, Tet repressor and MerR regulatory proteins as the sensing elements, for the detection of tetracycline and the toxic mercury in cell-free systems.^382^ Davies et al. constructed a cell-free protein array to screen high-immunogenicity proteins in human serums after virus infections, for the prophylactic uses and diagnosis.^383^ In remote regions or harsh environments, cell-free systems lyophilized and attached on papers (or other matrices) are convenient and stable.^384^ Pardee et al. employed lyophilized cell-free sensors to rapid determination of Ebola and Zika virus.^385,386^ Future cell-free synthetic biology may lead to sophisticated design and synthesis of more complicated therapeutic agents, or rapid and sensitive biosensors for chronic disease diagnostics.

## Discussion and future perspectives

Since the rapid developments started from more than a decade ago, synthetic biology has grown substantially and has emerged with many achievements, both in science and application aspects (Fig. 1). In this review, we summarized the advanced strategies and designs in synthetic biology for traditional pharmaceutical and medical applications, such as engineered smart cells (Fig. 2),^387^ live probiotic therapeutics,^151^ diagnostics,^388^ stem cells,^83^ drug production,^23^ nanocarriers^389^ and artificial vaccine developments.^300^ The novel approach will enrich clinical regimens, shorten drug development cycle and lower pharmaceutical prices.

Synthetic biology approaches that most probably bring (or has brought) dramatic changes in biomedical fields include: the use of light for time-spartial controllable precise cell therapeutics (optigenetics), designed bacteria to target cancer cells, engineered cells rewiring metabolic flux in human or engineer the gut-brain-liver axis (engineered live therapeutics). Recent studies have shown possibilities that biosystems mentioned above are functioning well in manmalian and exhibiting considerable therapeutic effects in animal models or even volunteers.^70^ However, they are just developed in their early stages. Many efforts are still needed to translate the lab findings to commercial products for patients.

The personalized engineered medicine is the next-gneration treatment strategy in the future. Smart therapeutics based on genetic-encoded circuits that can intepret environmental signal into effector activities will be commonly used. The auto-regulated therapeutic cells that sense diagnostic inputs for therapeutic outputs are one-station solutions for diagnosis, disease prevention and treatments (Fig. 2). Some applictions like CAR-T therapies have entered clinical stages, but most of the smart cells are not. Many attempts have failed in the early clinical, mainly for the low therapeutical abilities and unexpected side effects in human. Future works should emphasis on their safety as well as the efficacy and stability in treatments.

The combination of synthetic biology and artificial intelligence (AI) is promising to accelerate the advances both in medical and pharmaceutical fields, although the field is in initial stage. AI is a hit not only in computer science, but also in biology research.^390^ The AI prediction of protein structures ranks as the top one in ten scientific breakthroughs in 2021.^391^ The era of AI and big data is arriving, in-depth learning technique is advantageous in the characterization of complex objects,^392^ fusion of multimodal features^393^ and auto-sample generations.^394^ AI can be applied in the synthetic biology field. At present, the combined applications of AI and synthetic biology have mainly been focused on the following three aspects, including, firstly, foresight of future research directions; collection of related synthetic biology data, then distinguish the casual link to analyze and evaluate the application and development directions. This is very helpful in analysis of numerous clinical datasets. Secondly, in the pharmaceutical applications, screening effective drugs based on AI and bioinformatic big data, testing candidate chemicals and simulating the therapeutic processes in disease models. It is a high-throughput method saving much manpower. Thirdly, development of novel drugs via reconstruction or modification the genomes by in-depth AI learning models, synthesizing novel compounds for drug discoveries. In the future, AI is promising to assist medical synthetic biology in designing more complicated systems (engineered cells or tissues) based on actual demands, substantially decreasing labor amounts of researchers.

**Fig. 7:**
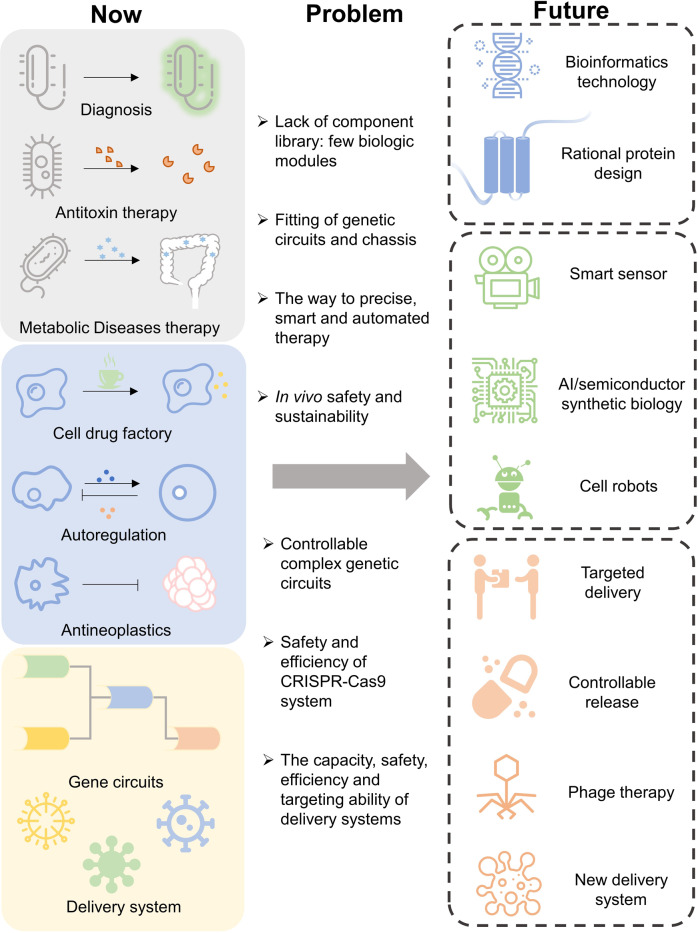
The present situations, technical bottlenecks and future developments of synthetic biology based gene therapies. Some diagnosis and therapeutical approaches are available via rewiring metabolic and (or) signaling pathways in present synthetic biology. However, some bottlenecks like safety, versatility and efficacy are needing to tackle. Besides, novel designs such as AI-aided synthetic biology and rationally constructed live organisms and proteins are progressing

However, some shortages and bottlenecks are to tackle for medical synthetic biology. Much effort is needed before the synthetic biology-based therapy become an available clinical option (Fig. 7). Although engineered cells containing genetic circuits are one of the most exciting designs in recent decades, they have limitations in actual uses of extracellular, signal-transduction free diseases which can be treated via traditional ways.^395^ Tissue-specific engineered therapeutics are not succeed till now. The interferences of manmalian metabolisms are remain unknown. Solving these problems will be helpful for synthetic biology-based clinical applications.

The majority of synthetic biology is still applied in microbes. However, most of the major issues, especially in solving human health problems, are needed for mammalian systems. Therefore, much efforts must be made for advancing mammalian synthetic biology to the next-generation therapeutic treatments, including the engineering of synthetic gene networks for disease treatments, tissue engineering or stem-cell generation and differentiation.

Additionally, synthetic biology-based therapeutics are still facing same social problems in ethical and legal fields similar to transgenic foods and stem cell therapies, although they can be imposed of better control from stringent pathways.

Even so, the future for synthetic biology-based therapeutics are promising, with new tools and applications developed in biomedical fields and highly-efficient microbial pharmaceutical production in the twenty-first century.
